# Sensing the Action: Rethinking Sensor Modalities and Multi-Modal Fusion in Vision–Language–Action Models for Robotic Manipulation

**DOI:** 10.3390/s26113541

**Published:** 2026-06-03

**Authors:** Byoung Chul Ko

**Affiliations:** Department of Computer Engineering, Keimyung University, Daegu 42601, Republic of Korea; niceko@kmu.ac.kr; Tel.: +82-10-3559-4564

**Keywords:** Vision–Language–Action, Sensor–Fusion–Action, robotic manipulation, sensor fusion, action representation, evaluation protocol

## Abstract

Recent Vision–Language–Action (VLA) models have rapidly emerged as general-purpose robotic policies that integrate language understanding, visual perception, and robot control. However, prior studies and surveys have primarily emphasized backbone architectures, action decoders, training recipes, and benchmark performance, whereas relatively limited systematic attention has been given to sensor modality selection, heterogeneous signal alignment and fusion, and their connection to action generation, all of which are critical to the performance and safety of real-world robotic manipulation. This survey addresses this gap by reinterpreting VLA within the framework of a sensor–fusion–action pipeline. This study first presents a systematic taxonomy of major sensor modalities, including RGB, depth, tactile sensing, force/torque, proprioception and inertial measurement unit, multi-spectral/thermal, and event-based vision, and compares them in terms of the physical information they provide, their characteristic failure modes, and their deployment constraints. This survey further reviews teleoperation-, human video-, and simulation-based data collection pipelines, together with representative dataset configurations, and analyzes the multi-modal design space from a sensor-centric perspective, including early and late fusion, cross-attention, token-level fusion, adapters, mixture of experts, and multi-rate action representations. In addition, this study identifies a strong bias in existing benchmarks toward RGB-centric inputs and single success-rate metrics and emphasizes the need for a multidimensional evaluation framework incorporating robustness, worst-case performance, safety, latency, and efficiency. By shifting the focus away from a model-centric narrative and explicitly accounting for real-world sensor complexity, this survey seeks to establish a sensor-centered foundation for the next generation of Physical AI.

## 1. Introduction

Artificial intelligence (AI) refers to computational systems capable of performing tasks that typically require human intelligence, such as perception, reasoning, and decision making. Deep learning (DL), a subfield of AI, employs multi-layered neural networks to learn hierarchical representations directly from data and has become the dominant paradigm for visual perception, natural language understanding, and robot control. Building upon these foundations, recent interest in Physical AI has increased rapidly, prompting active research on extending the knowledge and reasoning capabilities accumulated by generative AI and large language models (LLMs) to real-world robotic perception, planning, and control. Within this trend, Vision–Language–Action (VLA) models aim to integrate visual perception, natural language understanding, and robot control into a unified policy or pipeline, enabling robots to interpret user instructions and execute manipulation behaviors in real environments.

**Figure 1:**
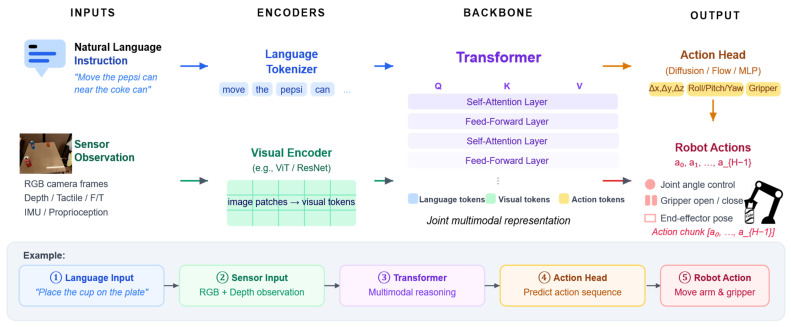
General architecture of a VLA model. Natural language instructions and visual observations are encoded separately and fused in a shared backbone, which generates robot actions through an action head. Depending on the policy design, the output may represent joint commands, end-effector targets, or short-horizon action chunks.

Figure 1 shows an overview of the general architecture of a VLA model. In a typical VLA framework, linguistic input and visual observations are encoded separately, processed jointly through a shared backbone, and finally translated into robot action sequences through an action head. This architecture highlights that VLA design is not merely a matter of input–output mapping; rather, it fundamentally depends on what sensor modalities are used, at what level heterogeneous signals are fused, and how actions are ultimately represented.

Over the past several years, VLA research has rapidly expanded under the broader vision of end-to-end robotic foundation policies. Viewed from a generational perspective, its development can be summarized as a stepwise progression: from behavioral cloning (BC)-based visuomotor mapping to integration with pre-trained Vision–Language Models (VLMs) and further toward general-purpose policies built on generative models. However, a common question has remained across all of these stages. Beyond scaling models and improving decoder heads, the issue of what the robot actually receives as input—that is, the choice of sensor modalities and the design of heterogeneous signal fusion—has remained insufficiently addressed, even though it is a critical factor governing both real-world performance and deployability.

**Figure 2:**
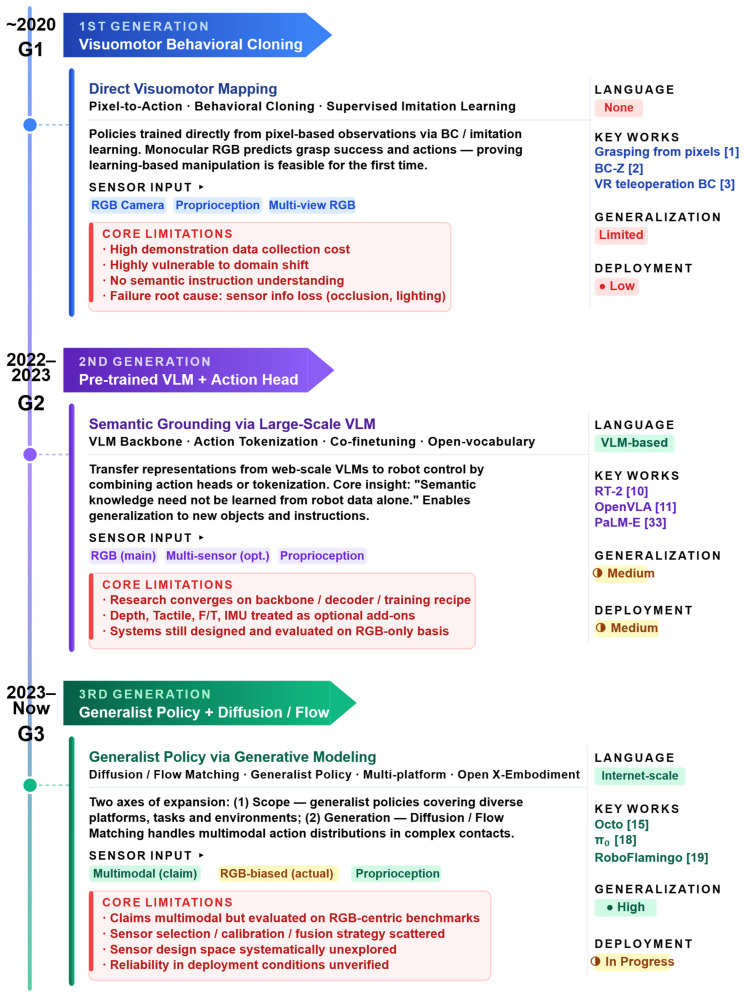
Three-generation evolution of VLA models. Each generation inherits sensor-design limitations from the previous one, a structural gap this survey directly addresses.

Accordingly, as illustrated in Figure 2, this survey examines the technological evolution of VLA models across generations and analyzes the structural sensing bottlenecks exposed at each stage of development.

### 1.1. 1st Generation

The first generation can be characterized as a visuomotor-mapping paradigm that preceded the full emergence of VLA. During this period, the dominant approach was to learn end-to-end policies that directly predicted actions from pixel-based observations using supervised learning or imitation learning. The key turning point of this generation was the reconceptualization of robotic manipulation as a problem that could be learned from large-scale demonstrations rather than engineered through rule-based pipelines. A representative example is Grasping from Pixels [1], which demonstrated the practical feasibility of pixel-to-action mapping by learning a policy that predicts grasp success directly from monocular RGB images through large-scale real-robot trials. Subsequently, BC-Z [2] extended this paradigm to large-scale behavioral cloning conditioned on language and video, whereas virtual reality (VR) teleoperation-based demonstration collection [3] enabled imitation learning for more complex manipulation tasks. However, these approaches still faced several fundamental limitations, including the high cost of data collection, vulnerability to domain shift, and the lack of semantic-level instruction understanding.

Robotic policies in this stage relied primarily on limited inputs, most commonly RGB cameras (sometimes in multi-view configurations) and proprioceptive signals. A substantial portion of failures arose not from insufficient model intelligence in itself but from information deficits induced by sensing limitations, such as occlusion [4], illumination changes [5], near-field distortion [6], and uncertainty in contact and friction [7,8]. In this sense, the first generation was highly significant as the starting point of robot learning; however, its input diversity and representational structure were fundamentally limited for achieving the robustness and safety required in real-world deployment.

### 1.2. 2nd Generation

The defining characteristic of the second generation lies in constructing VLA models by combining the semantic representations of pre-trained vision language models with an action head or decoder that outputs robot actions. The key turning point of this generation was the shift away from learning semantics solely from robot data. Earlier large-scale robot transformer policies such as RT-1 [9] provided an important precursor to this direction. By transferring the representational capabilities of vision language models trained on large-scale web data to robotics and by coupling them with action prediction heads or action tokenization mechanisms, researchers began to develop policies that could generalize to a wider range of instructions and situations. A representative example is RT-2 [10], which proposed expressing robot actions as text tokens and jointly fine-tuning them in the same format as a vision language model in order to connect internet-scale vision language pre-training knowledge to robot control. This approach demonstrated that the generalization ability and semantic reasoning capacity of robot policies could be extended to novel objects and language instructions. OpenVLA [11] further lowered the barriers to open access and reproducibility by introducing a 7B scale open-source VLA model and showed strong generalization performance and efficient fine-tuning based on large-scale real robot demonstrations.

Despite this rapid progress, the central focus of second-generation research tended to converge on questions such as which backbone, including VLMs or LLMs, should be used, how actions should be decoded through tokens or prediction heads, and which data mixtures and training recipes were most effective. As a result, many VLA systems continued to be designed and evaluated primarily around RGB-centric inputs, whereas heterogeneous sensor modalities such as depth, tactile sensing, force and torque, inertial measurement unit (IMU), and multi-spectral sensors were often treated as optional additions rather than core design elements. As this tendency carried over into the third generation, it became a more structural limitation of the field.

### 1.3. 3rd Generation

The third generation extends the previous stages along two main axes. The first is an expansion in scope, moving beyond single-task policies toward generalist policies that can operate across diverse platforms, tasks, and environments [12]. The second is an expansion in action generation, introducing generative models based on diffusion [13] or flow matching [14] in order to capture the multi-modal action distributions required for complex manipulation involving contact. For example, Octo [15] presents a general policy framework based on a transformer [16]-driven diffusion policy trained on large-scale robot data from Open X Embodiment [17] and demonstrates rapid adaptation across diverse observation and action spaces. π_0_ [18] combines a flow matching-based architecture with a pre-trained vision language model and proposes a direction for building generative policies that preserve internet-scale semantic knowledge while remaining suitable for robot control. RoboFlamingo [19] provides a relatively simple framework that connects an open vision language model such as OpenFlamingo [20] to robotic manipulation and reports competitive performance on language-conditioned manipulation even with limited fine-tuning.

Although the overall direction of this third-generation approach is appropriate, important parts of the design domain have not yet been sufficiently studied. In practice, although many studies claim multi-modality, they are still evaluated primarily on RGB-centric benchmarks, and strategies for sensor selection, calibration, and fusion are often scattered across individual system implementations rather than being treated as a central research topic.

Existing VLA surveys [21] also tend to organize the field around model architectures, tokenization schemes, training recipes, and benchmark performance, whereas sensor design and fusion strategies that directly affect deployment performance and safety receive relatively secondary attention. As a result, the field repeatedly encounters a structural limitation in which the assumption of RGB-based input cannot adequately explain common real-world challenges such as occlusion, low illumination, transparent or reflective objects, contact uncertainty, and issues of latency and synchronization. Overall, advancing VLA from laboratory demonstrations to real-world robotic manipulation requires not only larger models but also more effective sensing and more reliable fusion strategies.

Rather than centering on the dominant question of which model is better, this survey focuses on a more practical question: which sensors and fusion strategies determine the performance and safety of VLA systems. Here, a “sensor-centric perspective” refers to a design and evaluation approach in which sensor modality selection, physical measurement characteristics, noise and failure properties, and deployment constraints are treated as primary axes of analysis rather than as secondary implementation details. This perspective differs from a model-centric view, in which sensors are often assumed to provide reliable observations without being systematically analyzed as sources of uncertainty, latency, or system-level failure.

To address this question, this paper takes robotic manipulation as its primary domain, since it is the setting in which the influence of sensor design becomes most evident due to the need for precise state estimation and the presence of contact uncertainty. This survey aims to reframe the problem as a continuous design space that spans the physical limitations of sensor modalities, temporal and spatial fusion strategies, and constraint-based safety layers for real-world deployment. The specific contributions and organization of this paper are presented as follows:

**Literature search and selection scope.** This survey was conducted as a technical survey rather than as a PRISMA-style systematic review. The literature was identified through IEEE Xplore, ACM Digital Library, arXiv, Google Scholar, and publisher databases using combinations of terms such as Vision–Language–Action, VLA robot, robotic manipulation, multi-modal sensor fusion, tactile sensing, force torque sensing, RGB-D manipulation, event camera robotics, and robot policy evaluation. To improve reproducibility, representative Boolean-style search queries included the following: (“Vision-Language-Action” OR “VLA” OR “vision language action” OR “robotic foundation policy”) AND (“robotic manipulation” OR “manipulation policy”); (“multi-modal sensor fusion” OR “RGB-D” OR “tactile sensing” OR “force torque” OR “proprioception” OR “IMU” OR “event camera” OR “thermal sensing”) AND (“robotic manipulation” OR “robot policy”); (“action representation” OR “action tokenization” OR “diffusion policy” OR “flow matching” OR “action chunking”) AND (“Vision-Language-Action” OR “robotic manipulation”); and (“benchmark” OR “evaluation” OR “robustness” OR “latency” OR “safety”) AND (“VLA” OR “robotic manipulation”). The exact query syntax was adjusted as needed to match the search interface of each database. The search primarily covered publications from 2018 to 2025, with earlier foundational works included for sensor modeling, calibration, and robotic control. Papers were included when they addressed sensor modalities, multi-modal fusion, sensor-to-action data collection, action representation, or deployment-oriented evaluation in robotic manipulation. Works focused exclusively on non-manipulation tasks or perception-only benchmarks were excluded. The methodological scope and limitations of this technical survey, including the absence of PRISMA-style screening and independent dual-reviewer selection, are discussed in the dedicated section “Methodological Limitations of This Survey” presented immediately before the section “Conclusion”.

### 1.4. Related Surveys and Positioning of This Survey

Several recent surveys have reviewed VLA models and related embodied AI systems. Ma et al. [22] provide a broad embodied-AI taxonomy organized around model architectures and high-level task planners. Zhong et al. [23] center their analysis on action tokenization, whereas Shao et al. [24] and Li et al. [25] focus on large-VLM-based VLA architectures and training recipes. Din et al. [21] and Wang et al. [26] adopt a data-centric perspective, examining datasets, benchmarks, and data engines as the primary axis of analysis. Han et al. [27] provide the most fusion-oriented analysis among prior surveys but cover robotic vision broadly rather than VLA-specific manipulation, and address sensor failure modes and calibration only peripherally.

In contrast, the present survey explicitly adopts a sensor–fusion–action perspective. To support this positioning more concretely, this survey examines not only model architectures and training pipelines but also the sensor modalities used in representative VLA systems and datasets reviewed in Section 2 and Section 3. This analysis shows that RGB remains the dominant exteroceptive observation modality in current VLA research, whereas depth, tactile, force/torque, thermal, and event-based sensing are incorporated less consistently and are often treated as optional, task-specific, or system-dependent additions. This imbalance provides an evidence-based motivation for the structural RGB-centric bias discussed in this survey.

As summarized in Table 1, this work differentiates itself from prior surveys by systematically analyzing (i) the physical properties and failure characteristics of individual sensor modalities, (ii) calibration and synchronization as core components of the sensor-to-action pipeline, and (iii) sensor-centric multidimensional evaluation criteria. These dimensions have received limited systematic attention in prior VLA surveys. The comparison dimensions used in Table 1, including sensor modality taxonomy, failure mode analysis, calibration and synchronization, data collection, fusion strategy analysis, evaluation framework, and sensor-centric perspective, were selected as the primary axes along which sensor-centric survey coverage can be meaningfully differentiated. Each prior work was assessed according to whether the corresponding dimension is treated as a primary analytical focus, partially or peripherally addressed, or absent as a structured analysis axis.

### 1.5. Contributions and Organization of This Survey

This survey reconstructs VLA from the perspective of sensing, fusion, and action and makes the following six contributions:

- This survey provides a systematic taxonomy of the sensor modalities used in robotic manipulation, including RGB and multi-view cameras, depth sensors, tactile sensing, force and torque sensing, proprioception and inertial measurement units, and multi-spectral or thermal sensing. Rather than merely listing these modalities, this survey examines, for each modality, both the physical nature of the information it provides and the representative failure modes and hardware constraints that arise in real deployment settings, such as occlusion, low illumination, transparent or reflective objects, and contact uncertainty. In doing so, this survey argues in a systematic manner that sensor selection constitutes a fundamental starting point in VLA design.
- This survey provides analytical formulations for calibration-induced error propagation, temporal misalignment, modality information contribution, and latency effects. These formulations connect sensor-level design decisions to measurable outcomes in VLA-based manipulation.
- This survey systematizes the sensor to action pipeline through which sensor signals are transformed into actual policy inputs and action generation. To this end, this study reviews calibration and synchronization, data collection pipelines based on teleoperation, human video, and simulation, as well as methods for obtaining ground-truth labels tailored to manipulation tasks. This analysis shows that sensor design is not merely a matter of choosing input modalities but rather a practical pipeline problem that directly determines data quality, observation to action alignment, and ultimately the performance of the learned policy.
- This survey organizes the design space of fusion for aligning and integrating heterogeneous sensor signals across time and space from a sensor-centered rather than model-centered perspective. Representative strategies, including early fusion, late fusion, cross attention, and token-level fusion, are compared with particular attention to the trade-offs they present under latency, synchronization error, noise, and modality missingness. In this way, the survey moves beyond the question of which fusion method is best in general and instead provides design guidelines regarding which fusion strategy is appropriate under which conditions.
- This survey proposes an evaluation framework suited to sensor fusion-based VLA research. Most existing manipulation benchmarks are designed under the assumption of single RGB input and rely primarily on a single metric, namely task success rate. However, this metric does not capture the performance differences that occur when the sensor configuration changes. For example, depth sensors often fail in the presence of transparent objects, the absence of tactile sensing can lead to unstable grasping, and noise in IMU signals may result in delayed actions. To directly address these issues, this survey organizes a multidimensional set of evaluation criteria covering safety, robustness, real-time performance, and reproducibility and derives modality specific evaluation principles from the perspective of which metrics can properly reveal the contribution and failure characteristics of each sensor. Through this analysis, the survey seeks to connect the question of which sensors to use with the question of how to evaluate them under a coherent set of design principles.
- Building on the three preceding axes of analysis, this survey diagnoses the structural limitations caused by RGB-centric input bias and identifies open problems and future research directions for achieving deployment-level reliability. These include sensor agnostic VLA architectures, language grounding of tactile signals, theoretical frameworks for asynchronous multi-sensor fusion, and the absence of standardized benchmarks built on real sensor inputs.

The remainder of this paper is structured as follows: To make the scope of the survey explicit, the following guiding questions are addressed throughout the paper. **Q1:** Which sensor modalities are used in VLA-based robotic manipulation, and what physical information, failure modes, and deployment constraints does each modality introduce? **Q2:** How are heterogeneous sensor signals collected, calibrated, synchronized, and converted into policy-compatible observations? **Q3:** How are multi-modal sensor representations fused inside VLA architectures, and how do fusion strategies differ in terms of cross-modal interaction, latency, robustness, and computational cost? **Q4:** How does sensor design affect action representation, temporal alignment, and closed-loop execution? **Q5:** How should sensor-rich VLA systems be evaluated beyond task success rate, particularly in terms of robustness, safety, latency, efficiency, and deployment reliability? The remainder of this paper is structured according to these questions. Section 2 addresses Q1 by classifying the major sensor modalities used in VLA systems and examining the information each sensor provides together with its representative failure modes. Section 3 addresses Q2 by discussing the sensor-to-action pipeline through which sensor signals are converted into actual policy inputs via data collection, calibration, synchronization, and labeling processes. Section 4 addresses Q3 and Q4 by analyzing the design space for transforming heterogeneous sensor outputs into internal representations, integrating them through multi-modal fusion, and linking them to action representation and temporal alignment. Section 5 addresses Q5 by critically reviewing the limitations of existing benchmarks and evaluation protocols and proposing a sensor-centered multidimensional evaluation perspective. Section 6 outlines open problems and future directions related to deployment-level reliability. Section 7 discusses the methodological limitations of this survey. Finally, Section 8 summarizes the key message of this survey and emphasizes the importance of sensor-centric VLA research.

## 2. Sensor Modalities in VLA Systems

In real-world robotic manipulation, the performance and safety of VLA systems are not determined solely by the choice of model backbone or decoder. The physical signals that the robot observes, that is, the sensor modalities, and the way these signals are aligned across time and space and integrated into internal representations through multi-modal fusion directly affect policy success rate, out-of-distribution (OOD) robustness, real-time performance, and compliance with safety constraints. Nevertheless, existing VLA surveys and many empirical studies have largely treated sensor input as a default assumption, most often RGB, and have developed their discussions primarily around model design and training. To address this bias, this section classifies the sensors used in manipulation oriented VLA into four groups: vision sensors, tactile and force sensors, proprioceptive and IMU, and multi-spectral or thermal sensors. It then systematically examines the type of information provided by each modality, such as visibility, distance, contact, and inertia, together with their representative failure modes, including occlusion, reflection, synchronization error, and noise, as well as their deployment constraints, such as calibration, latency, power consumption, and durability. Through this analysis, this section argues that the question of which sensors to use is not merely a matter of hardware selection but a fundamental starting point for VLA design.

In multi-modal VLA systems, temporal alignment is a basic requirement for meaningful fusion. Because heterogeneous sensors operate at different rates and with different latencies, simple timestamp matching is often insufficient. In practice, alignment is typically handled through hardware synchronization when available and otherwise through timestamp-based offset correction, interpolation, buffering, and causal resampling. The implementation details are discussed in Section 3.1.

More formally, each sensor modality can be interpreted as a measurement process that maps an underlying physical state to an observable signal. Let $x_{t}$ denote the task-relevant physical state and $y_{t}^{\left(m\right)}$ the observation produced by modality mmm. A generic measurement model can be written as $y_{t}^{\left(m\right)}=h_{m}\left(x_{t}\right)+\epsilon_{t}^{\left(m\right)}$, where $h\left(\cdot \right)$ is the modality-specific measurement function and $\epsilon_{t}^{\left(m\right)}$ represents noise, bias, or unmodeled environmental disturbance. From this perspective, sensor selection in VLA is not merely a choice of input channels but a decision about which physical variables can be observed, with what uncertainty, bandwidth, latency, and failure modes.

### 2.1. Vision Sensors

Convolutional neural networks (CNNs) [28] are a class of deep neural networks specifically designed to process grid-structured data such as images. Each convolutional layer applies a set of learnable filters that slide across the input to extract local spatial features such as edges, textures, and parts, whereas pooling layers progressively reduce spatial resolution to capture larger receptive fields. By stacking multiple convolutional and pooling layers, CNNs build hierarchical feature representations in which lower layers capture low-level visual primitives and higher layers encode semantic concepts. This architectural inductive bias toward locality and translation invariance has made CNNs the dominant backbone for image classification, object detection, and feature extraction in robotic vision [28]. Beyond visual data, CNNs have also been adapted to process other sensor modalities, including inertial signals for motion and activity recognition [29], demonstrating their versatility across heterogeneous robotic perception tasks and robot-based industrial diagnostic systems [30]. Vision Transformers (ViTs) [31], in contrast, divide an image into fixed-size patches and apply self-attention mechanisms to capture global context across the entire image, offering strong scalability with large-scale pre-training data and reduced architectural inductive bias compared to CNNs.

However, vision sensors do not constitute a single homogeneous category. Rather, they include RGB cameras, depth or RGB-D sensors, event cameras, and fisheye or wide-angle cameras, each of which differs in physical sensing principle and output format. Therefore, instead of treating them as a single visual input, it is necessary to distinguish the type of information provided by each sensor, its role in VLA, and its limitations in real deployment.

**RGB Camera.** Monocular RGB cameras are, in practice, the de facto standard input in VLA research. A design in which camera frames are tokenized by a ViT [31] or ConvNet-based encoder [28], combined with language tokens for grounding, and then used to generate actions is among the simplest formulations and is also well suited to leveraging large-scale pre-training. The origins of this RGB-centric design can be traced back to large-scale behavioral cloning approaches developed before the full emergence of VLA. BC-Z [2] collected large-scale real manipulation tasks and combined RGB observations with language and video conditions, demonstrating zero-shot generalization to unseen tasks and showing that scaling with RGB-based supervision can provide a strong learning signal. SayCan [32] then marked an important transition in language-to-action integration by combining high-level plans proposed by a language model with visual low-level skills, represented as value functions or policies, in order to assess executability. PaLM-E [33] further extended this direction by integrating not only visual inputs but also continuous state inputs into the language model, thereby broadening the representation of robotic perception and state. Building on this line of work, RT-2 [10] experimentally showed that web-scale knowledge could be transferred to robot control through a recipe that jointly trained an RGB image-based vision language model together with robot action tokens. OpenVLA [11] proposed an open-source baseline that takes RGB observations and language instructions as input while emphasizing strong performance across diverse manipulation tasks and the practical efficiency of fine-tuning.

**Depth and RGB-D Sensor.** Depth sensors directly provide the three-dimensional geometric structure of a scene by measuring the distance value associated with each pixel. RGB-D cameras, such as Intel RealSense devices based on active stereo or structured light or Microsoft Azure Kinect based on time-of-flight sensing, output synchronized high-resolution RGB images and depth maps. The resulting point clouds serve as key inputs for grasp pose estimation and object shape recognition.

Recent work such as GR-1 [34] has shown that manipulation policies trained with RGB-D input can achieve grasping precision substantially superior to that of monocular RGB input. RoboAgent [35] improved the learning efficiency of a universal manipulation policy by transforming point clouds collected from multiple RGB-D cameras into an integrated 3D representation. Point Transformer [36] actively exploited 3D coordinates to define relations among tokens through neighborhood structures and relative positional encoding, whereas Point BERT [37] learned strong 3D representations through local patch tokenization and masked pre-training. In robotic manipulation, practical approaches such as PerAct [38] have also been developed, where 3D space is quantized into voxel grids to form token sequences.

The key strength of depth sensing is its ability to quantitatively measure the size, location, and shape of objects, enabling the creation of precise contact plans. However, depth sensors also exhibit structural limitations. For transparent objects, such as glass cups, or strongly reflective objects, such as metal bowls, infrared refraction and scattering can cause depth estimation to fail or produce severe noise. These conditions occur frequently in real kitchens and industrial environments and therefore constitute a major obstacle to reliable deployment of VLA systems in the real-world. In addition, time-of-flight (ToF)-based sensors are subject to limitations in measurable range and degradation in signal-to-noise ratio under strong ambient illumination, such as direct sunlight.

These limitations are not uniform across all depth sensors because different depth-sensing paradigms rely on different measurement models and therefore exhibit different error characteristics. Structured-light sensors project a known pattern onto the scene and recover depth from the deformation of the projected pattern. Their reliability is strongly affected by surface reflectance, ambient infrared illumination, and interference from other active projection sources. Stereo disparity methods estimate depth by triangulating matched points across two calibrated cameras, with depth uncertainty increasing approximately quadratically with distance: σz≈z2fbσd, where *f* is the focal length, *b* is the stereo baseline, and $\sigma_{d}$ is the disparity uncertainty. ToF sensors estimate depth from the round-trip time or phase shift of emitted light, and their dominant error sources include multipath interference, low surface reflectance, saturation, and motion artifacts, particularly on specular, translucent, or fast-moving objects. In practice, structured light is often effective at short range under controlled indoor conditions, stereo sensing is useful in mid- range environments when sufficient texture is available, and ToF sensing is less dependent on scene texture but can be limited by multipath effects and strong ambient infrared illumination. These distinctions directly affect which depth modality is appropriate for a given manipulation context.

**Event Camera/Dynamic Vision Sensor (DVS).** Unlike frame-based cameras, event cameras asynchronously output only the timestamp, spatial location, and polarity of brightness changes of each pixel [39]. Early DVS hardware reported latency at the microsecond scale, dynamic range exceeding 120 dB, and low power consumption, properties that provide a favorable physical basis for always on sensing in edge environments [40]. Event cameras can also provide useful visual signals in situations involving high-speed motion or abrupt illumination changes, where conventional frame cameras often lose information due to motion blur. Studies that reconstruct high-speed and high-dynamic-range images from event streams have empirically demonstrated these advantages [41].

However, the use of event cameras in the context of VLA remains at an early stage. Because event streams are sparse and asynchronous, conventional frame-based RGB processing pipelines cannot be applied directly. More importantly, the lack of large-scale labeled datasets and pre-training resources has repeatedly been identified as a major bottleneck [42,43]. To mitigate these limitations, recent work has begun to explore cross-modal alignment and pre-training transfer strategies that adapt the representations of vision language models trained on internet-scale RGB data to event-based representations. For example, attempts have been reported to transfer contrastive language-image pre-training (CLIP) [44] style representations to event vision [45]. This line of event-to-RGB knowledge transfer represents an important research opportunity for enabling future VLA systems to achieve human level responsiveness and robustness under fast and challenging real-world conditions.

**Fisheye and Wide-Angle Cameras.** Fisheye and wide-angle cameras provide a wide field of view with a single sensor, which makes them particularly advantageous in mobile manipulation scenarios where the robot must simultaneously perceive the surrounding environment of the base, the manipulation target, and the local scene near the end effector within a single observation. In long-horizon manipulation settings, such as narrow indoor spaces or complex household environments where scene context must be maintained continuously, a wide field of view can reduce blind spots and mitigate vulnerability to occlusion. This need is also indirectly reflected in large-scale robot data collection. For example, DROID [46] employs a multi-view stereo camera setup, including externally fixed viewpoints and wrist-mounted viewpoints, in order to ensure diversity in real-world manipulation data. Dobb-E [47] likewise demonstrates the practical utility of camera-based observations for everyday deployment by collecting and using RGB and depth video in home environments with a smartphone-based device while targeting on device real-time control.

At the same time, a wide field of view often introduces strong radial distortion and nonlinear projection, which can create a domain gap with ViT-based backbones pre-trained on perspective image distributions [31]. Simple rectification alone may still leave information loss or residual distortion. Accordingly, recent work has proposed single view calibration methods that are less dependent on a specific camera model, such as AnyCalib [6], as well as approaches that learn a general camera model even in environments where camera parameters are uncertain, such as AnyMap [48]. As a result, although wide-field-of-view cameras are being used increasingly in data collection and robotic systems, architectural and fusion strategies that directly learn from and integrate fisheye or wide-angle observations within VLA remain a largely underexplored design space.

Although all vision sensors fall under the common category of visual input, in practice they offer different strengths: RGB sensors are strong in semantic information, depth sensors in geometric information, event cameras in temporal resolution, and fisheye or wide-angle cameras in spatial coverage. Therefore, future VLA design should move beyond treating vision as a single modality and instead consider which visual sensor is needed under which manipulation conditions and how that sensor should be aligned with language and action representations.

Table 2 summarizes representative vision sensors in terms of their sensing role, typical use in VLA systems, and deployment limitations.

### 2.2. Tactile and Force Sensors

Vision sensors effectively capture object shape, arrangement, and semantic information, but they are inherently limited in observing physical contact states that are critical for precise manipulation, such as surface friction, elasticity, deformation, subtle slip, and the spatial distribution of contact forces. This limitation is particularly evident in grasping and in-hand manipulation. Even when a grasp appears successful in RGB observations, unstable force or shear distributions at the actual contact surface can still lead to slip and, ultimately, task failure. For this reason, tactile sensors and force/torque sensors should not be regarded as merely auxiliary inputs but rather as core feedback channels that directly influence performance and safety in contact-rich manipulation.

**Definitional clarification: tactile sensing vs. haptic sensing.** Although the terms tactile and haptic are sometimes used interchangeably in the broader robotics literature, they refer to distinct sensing paradigms. This survey uses tactile sensing to denote the autonomous acquisition of contact-related physical signals such as pressure, shear, vibration, and surface texture by sensors mounted on the robot itself, with the resulting signals consumed directly by the policy as feedback for closed-loop control. In contrast, haptic sensing or haptic feedback refers to systems in which contact information is rendered to a human operator through force-feedback devices, typically for teleoperation, rehabilitation, or shared-control applications [49,50]. Because this survey focuses on autonomous robotic manipulation in VLA systems, where the policy itself acts as the decision-making agent, our discussion is limited to tactile sensing in the former sense. Haptic feedback is mentioned only when relevant to teleoperation-based data collection (Section 3.2).

**Tactile Sensor.** The importance of tactile sensing is clearly demonstrated in grasp outcome prediction. Calandra et al. [51] showed that combining vision and tactile information significantly improves the grasp outcome prediction accuracy and real-robot grasp success rate compared with using visual information alone, thereby providing experimental evidence that visible and felt information are complementary in assessing grasp stability. In subsequent work, Calandra et al. [4] further showed that visuo-tactile policies incorporating tactile feedback can improve stability through repeated corrective behaviors such as regrasping, highlighting the importance of closed-loop strategies for mitigating subtle instabilities that arise after contact. These gains, however, are accompanied by additional computational and system-level costs. Adding tactile sensing to a vision-based manipulation system increases computational latency, data bandwidth, synchronization requirements, and sensor calibration burden, especially when image-based tactile sensors are used [29]. In contact-rich or safety-critical tasks, these costs may be justified by improved grasp stability and slip prevention, whereas in high-throughput manipulation of rigid and well-structured objects, the added complexity may outweigh the marginal performance gain.

From the perspective of VLA, one particularly important development is the image like output format of optical tactile sensors. GelSight [7] measures fine surface deformations at the contact interface as high-resolution images using a transparent elastomer together with internal illumination and a camera and can also provide cues about shear and slip through marker displacement, thereby enabling joint estimation of contact geometry- and force-related information. Dong et al. [52] improved GelSight-style sensors to measure contact surface shape and slip more reliably, showing that tactile-based contact state recognition can provide practically useful information within manipulation pipelines. DIGIT [53] further miniaturized and reduced the cost of the GelSight design, offering high-resolution tactile images in a form factor that can be mounted on the fingertip and applied to a wide range of contact tasks, including in-hand manipulation. This image-based output format of tactile sensing makes it easier to directly reuse CNN- or ViT-based visual encoders, as well as tokenization- and attention-based fusion designs similar to those used for RGB input, thereby lowering the barrier to integrating tactile sensing into VLA. More recently, TLA [54] has introduced a Tactile Language Action model that integrates tactile signals together with language conditions for manipulation, exploring how contact information can contribute to interpreting the physical meaning of instructions such as gently, firmly, or without slipping and to generating appropriate actions. In addition, approaches that incorporate tactile feedback into existing VLA pipelines to improve execution time stability, such as slip detection or contact anomaly handling, have also been reported, suggesting that tactile sensing is increasingly serving not only as a training input but also as a source of safety feedback during execution [55].

The measurement chain of GelSight-style optical tactile sensors can be described in three stages. First, contact force and local contact geometry deform the elastomer surface. For small deformations, this deformation can be locally approximated by a calibration-dependent compliance relationship, u≈Cp, where *u* denotes surface displacement, *p* denotes applied pressure, and *C* is an effective compliance parameter determined by the elastomer material, thickness, and boundary conditions. Second, the deformed surface is imaged under controlled internal illumination. In many GelSight-style sensors, surface normals are estimated using photometric stereo, and the normal field is then integrated to reconstruct a contact height map. Third, when embedded markers are used, shear deformation and slip can be estimated by tracking marker displacement over successive frames. The overall pressure-to-image mapping is therefore nonlinear, material dependent, and calibration dependent. Consequently, contact force estimation from tactile images may exhibit systematic bias when the contact geometry, friction condition, loading range, or sensor state differs from the calibration setting, which is a deployment constraint directly relevant to real-world manipulation.

**Force/Torque Sensors.** Force/torque (F/T) sensors are typically mounted at the robot wrist and measure, in real-time, the six-axis forces and torques exerted by or applied to the end effector. In tasks such as peg-in-hole assembly, door opening, insertion, and deformable object manipulation, where contact is frequent and force regulation is essential, F/T-based compliance and impedance control play a decisive role in both success rate and safety. From the viewpoint of VLA, an important observation is that language instructions often convey not only task goals but also force related constraints and preferences, such as strongly, gently, or stop when resistance increases. Accordingly, force torque signals provide critical evidence for detecting hazardous situations such as excessive contact force or jamming, for setting control parameters such as force thresholds and compliance direction or stiffness, and for diagnosing the causes of failure. At present, however, VLA systems that fully integrate language conditioning with F/T-based control at scale remain limited. More robust progress has instead been made in contact-rich imitation learning and control frameworks that explicitly incorporate force signals. For example, ForceMimic [56] combines force motion capture-based demonstration collection with hybrid force position control primitives for force-centric imitation learning, thereby moving force signals to the center of learning for strongly contact-driven manipulation. Rather than placing language integration at the forefront, such approaches first establish a reliable foundation for learning and controlling force and contact. Once integration with language conditions is built on top of this foundation, force torque signals can serve as essential cues for threshold setting, compliance direction selection, and failure diagnosis. Nevertheless, achieving such integration requires solving several system level design challenges in advance.

The practical challenge of integrating tactile and force sensing into VLA lies not simply in adding new sensors but in reliably aligning signals that differ in sampling rate and latency through multi-rate alignment, designing asynchronous triggers based on tactile events such as slip cues, and hierarchically separating low-level control loops for force stabilization from high-level policies for language-conditioned planning. In addition, tactile sensors are highly susceptible to domain shift caused by durability issues, contamination, and material aging, which makes calibration and robustness enhancement essential requirements for long-term deployment.

**Performance trade-off and tolerability of additional sensing sources.** In this survey, the performance affected by adding sensing sources is not limited to task success rate alone. It should be interpreted as a multidimensional, deployment-oriented profile that includes task success rate, grasp stability, robustness to sensor noise or missing modalities, safety-related events such as excessive contact force or slip, inference latency, policy-loop latency, and compute cost, and sensor contribution measured through ablation. From this perspective, the computational overhead introduced by additional sensors should be regarded as tolerable only when two conditions are jointly satisfied: first, the end-to-end policy-loop latency remains within the control budget required by the task; and second, the additional modality provides a measurable gain in robustness, safety, or execution quality that cannot be obtained reliably from the existing sensor set alone. Formally, a sensing modality *m* can be considered tolerable when T_loop_ + ΔT_m_ ≤ T_budget_ and ΔP_m_ ≥ δ_P_, where ΔT_m_ is the increase in end-to-end policy-loop latency, T_budget_ is the task-specific control budget, ΔP_m_ is the gain on the target deployment criterion (for example, success rate, reduction in slip or collision events, or reduction in force violations), and δ_P_ is the minimum performance gain required for the target application.

This trade-off is inherently task dependent, as summarized in Table 3. In contact-rich manipulation, such as insertion, peg-in-hole assembly, deformable-object handling, or grasp stabilization, the additional cost of tactile or force/torque sensing is often justified because these sensors provide direct evidence of contact, slip, resistance, and excessive force, which cannot be recovered reliably from vision alone. In contrast, for high-throughput manipulation of rigid, visually well-observed objects, the same sensing overhead may be negligible only if the additional signals are processed at low cost and may not be justified at all if it increases synchronization and inference latency without improving success or safety. Related real-time sensing studies in inertial-sensor and exoskeleton-based activity recognition similarly show that sensor configuration should be evaluated jointly in terms of recognition performance and computational time, although these studies fall outside the strict scope of VLA-based manipulation policies [57,58]. The corresponding evaluation metrics that make this trade-off measurable are discussed in Section 5.

### 2.3. Proprioceptive Sensors (IMU; Joint Encoders)

Proprioception serves as the primary feedback channel for the robot’s own instantaneous pose and internal state, complementing vision and tactile sensing, which mainly observe the external environment. These signals are not affected by illumination changes or occlusion, and in control loops they are often measured at high frequencies ranging from several hundred Hz to kHz. However, VLA policies during training and inference typically operate at only a few to several tens of Hz. As a result, practical systems must resample proprioceptive streams to match the policy frequency and temporally align them with visual and language inputs. In addition to robustness against illumination changes and occlusion, another important advantage of IMU-based sensing is portability. Compared with external optical motion-capture systems or fixed camera installations, IMUs are compact, low cost, wearable or easily attachable to robot links, and can be deployed outside controlled laboratory environments. This portability has made IMUs particularly useful in clinical and wearable motion-analysis applications, including continuous human activity recognition and pathological gait recognition, where long-term monitoring and unconstrained measurement are often required [57]. Although these clinical studies are outside the strict scope of VLA-based robotic manipulation, they provide useful evidence that inertial sensing can serve as a practical alternative or complement to optical sensing when portability, workspace independence, and deployment flexibility are important.

IMU measurements are commonly modeled as angular velocity and linear acceleration corrupted by additive noise and slowly varying bias drift. For example, gyroscope measurements can be written as ${\tilde{w}}_{t}=w_{t}+b_{t}^{w}+\eta_{t}^{w}$, whereas accelerometer measurements can be expressed as ${\tilde{a}}_{t}=R_{t}^{T}\left(a_{t}-g\right)+b_{t}^{a}+\eta_{t}^{a}$, where ${\tilde{w}}_{t}$ and ${\tilde{a}}_{t}$ denote the sensor-measured angular velocity and linear acceleration, and $w_{t}$ and $a_{t}$ denote the corresponding true values. In addition, $b_{t}^{w}$ and $b_{t}^{a}$ denote bias terms, and $\eta_{t}^{w}\;$ and $\eta_{t}^{a}$ denote measurement noise. Since VLA policies typically operate at a much lower frequency than IMUs, high-rate inertial streams are often filtered, downsampled, summarized, or pre-integrated before being used by the policy. These operations can accumulate noise and bias-related uncertainty over time, making IMU reliability dependent on the integration window, calibration quality, and correction from other sensing modalities. This issue is directly relevant to replanning frequency design and the reliability-weighting strategies discussed in Section 4.2.

**Connecting robot self-state estimation to the VLA action head.** In both early manipulation policies and recent VLA systems, proprioception is typically represented as a low-dimensional state vector, such as joint states or end-effector states (the latter being a parameterization of T∈SE(3)), and used as a conditioning input together with visual tokens. For example, ACT [59] generates future action sequences in chunk units conditioned on multi-camera observations and current joint states, thereby providing a practical policy form that mitigates error accumulation over long-horizons. Similarly, diffusion policy [13] conditions action generation jointly on RGB observations and low-dimensional robot states, such as joint or end-effector states, thereby emphasizing the role of state feedback in precise control regimes where visual information alone may be insufficiently stable.

In recent generalist VLA systems, the standardization and tokenization of proprioceptive input have become even more important because a single policy is expected to operate across heterogeneous robots and tasks. Open X-Embodiment [17], which integrates manipulation datasets from 22 robots across 21 institutions, makes clear that the normalization and standardization of states and actions are essential preprocessing steps for generalist learning when different robots have different degrees of freedom and control interfaces. OpenVLA [11] proposes a framework that predicts tokenized output actions from robot observations and then decodes them into continuous control signals, thereby suggesting a path for transferring policies learned from large-scale manipulation data across diverse robotic platforms. RT-2 [10] likewise attempted to combine web-scale pre-training with robot control by representing robot actions as text-like tokens that match the output format of a vision language model.

**Figure 3:**
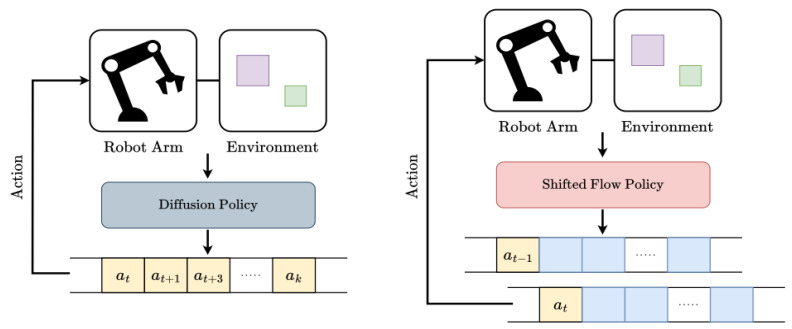
Comparison between action chunking and the proposed shifted flow. (**Left**) Action chunking predicts a fixed-length sequence of actions that all share the same timestep and are executed in an open-loop manner by relying on a single observation. In contrast, (**Right**) shifted flow assigns linearly increasing timesteps to each action within the prediction window to model future uncertainty by enabling updated observations to condition each sampling step (this image is adapted from [61]).

**Feedback Loop Design for End-effector Pose.** When proprioception is viewed not merely as a state input but as a practical factor governing the stability of closed-loop control, multi-rate design emerges as a central issue. Because joint and IMU signals are acquired at high frequencies whereas policy inference is typically performed at a much lower rate, practical systems often adopt strategies such as (i) predicting actions in delta form so that accumulated errors can be corrected progressively or (ii) maintaining a buffer of future action sequences and updating it at every step. For example, streaming diffusion policy (SDP) [60] improves responsiveness while reducing inference cost by reusing and updating previously predicted, partially denoised future action sequences. By contrast, action chunking-based policies [59] predict multiple future actions from a single observation in an open-loop manner, which creates a limitation in that proprioceptive feedback updated during execution cannot be reflected in action generation. Shifted Flow Policy (SFP) [61] addresses this limitation by introducing a reparameterization that linearly shifts the time step according to the action index, thereby enabling a closed-loop structure in which actions are generated at each sampling step conditioned on updated observations as shown in Figure 3. In this way, SFP improves the real-time incorporation of proprioceptive feedback and alleviates the open-loop limitation of action chunking. This line of work suggests that, independently of which model architecture is used, system design for aligning proprioceptive streams with the policy frequency has a direct impact on manipulation performance and safety.

### 2.4. Multi-Spectral/Thermal Sensors

Multi-spectral, thermal, and infrared sensors remain underexplored in manipulation-oriented VLA systems, despite their importance in industrial and safety-critical environments. Nevertheless, for robots to operate safely and reliably in industrial environments, such as welding, casting or sintering, handling of high-temperature objects, and equipment inspection, thermal states such as temperature and heat distribution, as well as visibility and material cues under dust or smoke, become critically important, since these cannot be adequately captured by visible spectrum RGB alone.

From a physical perspective, long-wave and mid-wave infrared (LWIR/MWIR) sensing measures the radiance emitted by an object and estimates the surface temperature distribution under calibrated conditions, whereas multi-spectral sensing based on near-infrared and short-wave infrared (NIR/SWIR) primarily decomposes the reflectance properties of object surfaces across multiple wavelength bands under external illumination, thereby providing cues about material properties, moisture, contamination, and visual contrast in smoky or dusty environments. Thermal sensors have strong potential to provide semantic cues directly relevant to manipulation. For example, LWIR cameras, typically operating in the 8 to 14 μm range, can support judgments such as whether an object is too hot to grasp, whether a material has not yet solidified, or whether there is an abnormal heating pattern that may indicate equipment failure, based on surface temperature gradients, overheated regions, and anomalous heat distributions. Multi-spectral sensors can also play an important role in manipulation. In particular, NIR and SWIR bands can be advantageous under conditions in which scattering effects are reduced compared with visible light, such as smoke, light fog, rain, or humid environments. Indeed, prior studies have quantitatively shown that SWIR imaging can provide more robust observations than visible spectrum imaging under foggy and rainy conditions.

Despite this potential, thermal and multi-spectral sensors have not yet been fully integrated into VLA systems, and several structural barriers remain. First, there is a severe shortage of large-scale thermal language and multi-spectral language paired data suitable for pre-training. This limitation is not merely a matter of data scale but also reflects a representation gap arising from the mismatch between tasks such as temperature reasoning and thermal semantic understanding and the learning biases of RGB-based vision language models. Recent benchmarks such as ThermEval [62] show that although vision language models may perform reasonably well at distinguishing modalities in thermal images, their performance deteriorates substantially on tasks involving temperature reasoning or estimation, underscoring that thermal reasoning remains an open problem.

Second, the sensors themselves also impose substantial constraints. LWIR sensors often have limited spatial resolution and frame rate, require non-uniformity correction and continued calibration maintenance, and, most importantly, may produce different observed values even at the same true temperature because of emissivity differences, which necessitates additional calibration for accurate temperature estimation [63].

Third, the alignment of intrinsic and extrinsic parameters and temporal synchronization across RGB, thermal, and possibly depth sensors, as well as policy-level fusion design including early fusion, late fusion, cross attention, and token-level fusion, has not yet been standardized. As a result, although these sensors may appear beneficial when simply added to a system, they are difficult to connect to reproducible training and evaluation protocols within actual VLA pipelines. Future research should address these challenges in order to move beyond RGB bias and extend VLA toward the level of reliability required for industrial deployment.

## 3. Sensor-to-Action Pipeline

This section focuses on how heterogeneous sensor signals are collected, aligned, and validated before being transformed into inputs for VLA training. Specifically, this section reviews calibration and synchronization, data collection pipelines, and ground-truth annotation methodologies tailored to manipulation tasks in sequence.

### 3.1. Calibration and Synchronization

For the fragmented sensor signals collected by a VLA system to be translated into precise robotic action generation, it is essential to align heterogeneous data within a unified spatiotemporal context. In VLA systems designed for high-precision manipulation, calibration and synchronization are not merely engineering level preprocessing steps but a technical foundation for preserving the causal integrity of the observation–action pairs on which the model is trained. If spatial misalignment or temporal delay across sensors is not properly corrected, the resulting causal corruption in the training data may directly lead to control failure or unsafe behavior in real deployment. Accordingly, this section systematically examines the major strategies and underlying necessity of spatial extrinsic calibration, temporal synchronization, and hand–eye alignment, which together serve as key gateways that determine both the quality of VLA data and the precision of execution.

**Spatial calibration.** In multi-camera systems, extrinsic calibration estimates the rigid-body transformation between camera coordinate frames. Let $T_{a\leftarrow b}\in SE(3)$ denote the transformation that maps a point from frame *b* to frame *a*. This transformation is represented as a homogeneous transformation matrix, where the rotation $R_{a\leftarrow b}\in SE(3)$ and translation $t_{a\leftarrow b}\in R^{3}$ are jointly encoded as

(1) $$T_{a\leftarrow b}=\left[\begin{array}{cc} R_{a\leftarrow b} & t_{a\leftarrow b}\\ 0^{T} & 1 \end{array}\right]\in SE(3)$$

A point $p_{b}$ expressed in frame *b* is mapped to frame aaa as ${\tilde{p}}_{a}=T_{a\leftarrow \beta }{\tilde{p}}_{\beta }$ in homogeneous coordinates, where ${\tilde{p}}_{a}={\left[p_{b}^{⊺},1\right]}^{⊺}$ [64,65]. This formulation enables observations from different viewpoints to be integrated into a consistent coordinate frame.

Conversely, errors in the estimated $T_{a\leftarrow b}$ lead to poor point cloud alignment and errors in object position or pose estimation, which ultimately propagate into grasp pose errors. In particular, for a manipulation distance *d*, a rotation error *δθ* can induce a positional error on the order of δθ·d·tan(δθ). Therefore, even small angular errors cannot be neglected in millimeter-to-centimeter-scale precision manipulation.

From this perspective, a representative approach for jointly estimating the spatial and temporal parameters of an entire sensor array is spatiotemporal calibration based on continuous time estimation. Furgale et al. [66] proposed a framework that jointly estimates both the time offset and the spatial displacement among heterogeneous sensors. Taylor and Nieto [67] further systematized a motion-based method for simultaneously estimating multi-modal sensor extrinsics and timing offsets, thereby providing a calibration perspective applicable even to sensor arrays composed of heterogeneous hardware.

**Temporal synchronization.** In multi-modal sensing systems, temporal alignment is as important as spatial alignment. RGB cameras typically operate at tens of Hz, depth sensors at similar rates, joint encoders at kHz, and IMUs at hundreds of Hz to kHz, each with different sampling periods and latencies. If one relies only on software timestamps, a nontrivial temporal offset may remain in the observation–action matching process. In practice, resampling is often a necessary first step for aligning high-rate streams, such as IMU or proprioceptive signals, to the lower policy frequency. However, resampling alone may still be insufficient when unknown sensor offsets or hardware-dependent latencies remain, in which case-explicit offset estimation or causal reconstruction of policy inputs under a multi-rate structure is additionally required. Even a small residual temporal offset Δ*t* can induce non-negligible pose mismatch during manipulation, approximately proportional to the motion magnitude over that interval; thus, if the robot or object state changes rapidly, timestamp matching and resampling alone may not preserve correct observation–action alignment. As emphasized by Taylor and Nieto [67], approaches that estimate timing offsets jointly with extrinsics are particularly useful under field conditions. For the robot–camera time offset problem that frequently arises in robotic manipulation, Koide and Menegatti [68] presented a synchronization method that estimates the temporal offset between robot pose trajectories and camera observations by minimizing reprojection error.

From a system-integration perspective, reliable temporal synchronization requires not only algorithmic offset estimation but also appropriate middleware, clock synchronization, and hardware-level support. In ROS2-based systems, the data distribution service (DDS) communication layer provides configurable Quality-of-Service policies, such as reliability, history depth, deadline, and durability, which affect sensor topic delivery, buffering, and real-time data availability. However, software-level timestamping and message transport can still introduce non-negligible jitter under computational or communication load. Hardware-level synchronization protocols, such as such as the Precision Time Protocol (PTP; IEEE 1588-2008/2019), can reduce inter-device timing error to microsecond or sub-microsecond levels when hardware timestamping and suitable network support are available. Therefore, a hardware abstraction layer that exposes a unified timestamping interface across heterogeneous sensors, including cameras, IMUs, F/T sensors, and tactile arrays, is an important infrastructure component for VLA data collection and deployment. Such an interface helps maintain temporally consistent observation–action pairs even when the underlying sensors differ in driver, sampling rate, and communication protocol. In practice, many robotic platforms further improve synchronization by using hardware trigger signals from a central controller to align camera exposure, IMU sampling, and robot state logging within a shared clock domain.

Beyond infrastructure-level synchronization, multi-rate sensor streams require algorithmic treatment to maintain temporal consistency. Naive downsampling of high-frequency signals, such as decimating a 1 kHz IMU stream to 10 Hz for policy input, can introduce aliasing when the signal contains components above the Nyquist frequency of the target rate. Therefore, low-pass anti-aliasing filtering should be applied before decimation. When multiple sensors must be fused into a temporally consistent state estimate, two representative frameworks are particularly relevant. In Kalman-filter-based fusion, the state estimate is propagated using a high-rate process model, such as IMU integration, and corrected whenever lower-rate measurements, such as RGB- or depth-based pose observations, become available. This naturally supports asynchronous updates as long as reliable timestamps are available. In factor-graph-based formulations, state variables at different timestamps are represented as nodes, whereas sensor measurements are represented as factors that constrain the trajectory. The joint state trajectory can then be estimated through maximum a posteriori inference. This formulation is especially useful for offline calibration, post hoc dataset labeling, and multi-modal trajectory reconstruction because it can incorporate measurements acquired at irregular timestamps. For VLA data collection, the practical implication is that raw sensor logs should be preserved at their native rates with hardware timestamps whenever possible, whereas rate conversion and fusion should be applied as post-processing steps so that the fusion strategy can be adapted without recollecting data.

The effects of spatial and temporal misalignment can be further interpreted from an error-propagation perspective. A residual translation error δp and rotation error δθ in extrinsic calibration can induce a position error approximated as $\left\|\delta x\right\|\approx \left\|\delta p\right\|+d\cdot \left\|\delta \theta \right\|$, where *d* denotes the sensor-to-target distance. Similarly, a temporal offset ∆t combined with end-effector velocity $v_{t}$ can produce a state mismatch approximated as $\left\|\delta x_{t}\right\|\approx \left\|v_{t}\right\|∆t$. These relationships explain why high-precision manipulation requires accurate spatial calibration and tight temporal synchronization, particularly when millimeter-level pose accuracy or rapid contact response is required.

**Hand–eye/robot–world calibration.** In eye-in-hand settings, where the camera is mounted on the wrist, and eye-to-hand settings, where the camera is externally fixed, hand–eye calibration determines the transformation between the camera coordinate frame and the robot kinematic frame. If this transformation is inaccurate, a systematic error remains such that the robot may fail to reach the intended target location even when the target is visually estimated correctly. Moreover, if hand–eye calibration is inaccurate during demonstration collection, the training data themselves accumulate as misaligned visual–action pairs, thereby limiting the upper bound of policy performance.

For this problem, Koide and Menegatti [69] proposed a hand–eye calibration method that directly minimizes reprojection error without explicitly estimating camera pose and demonstrated its extensibility to a variety of camera models. Going further, Junhyoung Ha [70] presented a probabilistic framework for hand–eye and robot–world calibration in the form of AX=YB, showing that measurement noise models and differences in reliability can be incorporated in a systematic manner.

Because calibration may drift in real deployment as environments change, recent work has also explored learning-based approaches that estimate hand–eye transformations without requiring separate calibration objects. For example, Pachtrachai et al. [71] proposed a method for estimating hand–eye transformations without a calibration grid and discussed its applicability in field settings where resetting costs are high. More broadly, calibration should be treated not only as a one-time preprocessing step but also as a compensatory stage within the data pipeline. In practice, many robotic systems benefit from an automatic preliminary calibration or refinement stage before large-scale data collection, together with reprojection-error-based correction, motion-based estimation of spatial and temporal offsets, and target-free recalibration when the environment or hardware configuration changes [66,67,69,71]. The automation and robustness of such calibration procedures are therefore essential to building self-improving data pipelines for large-scale VLA training.

### 3.2. Data Collection Pipelines for VLA Training

Multi-modal data collection in VLA systems should be understood as a sensor-to-action design problem rather than merely as a process of recording robot demonstrations. It determines which sensor streams are acquired, how cameras and other sensors are spatially arranged, how observation–action pairs are synchronized, and how occlusion, calibration noise, and deployment bias enter the training data. From this perspective, existing VLA data pipelines can be broadly organized into three categories: (A) robot teleoperation and demonstration-based collection, (B) indirect use of human video and web-scale data, and (C) simulation-based collection with data generation and augmentation.

**Teleoperation and robot demonstration.** From a sensor-centered perspective, teleoperation is important because it defines how multi-modal observations and robot actions are coupled during data collection. The most canonical approach is to record demonstrations while a human operator remotely controls the robot. For example, ALOHA [59] showed that bimanual manipulation demonstrations can be collected with relatively low-cost hardware, and emphasized the importance of multi-camera configurations, such as a fixed workspace view together with wrist or close-range views, for stably observing the workspace. BridgeData V2 [72] released a large-scale set of manipulation trajectories based on low-cost robot arms and systematically demonstrated how diverse environments, camera poses, and task conditions affect data diversity and generalization. DROID [46] is a large-scale teleoperation dataset composed of more than 76,000 demonstration trajectories collected across 52 buildings and 564 scenes and reported that the consistency of multi-camera extrinsic calibration protocols is a key factor determining the quality of data integration across heterogeneous environments. Dobb-E [47] proposed collecting RGB and depth video in household environments using a smartphone-based device, thereby showing the feasibility of acquiring in-the-wild data with diverse sensor configurations even without specialized hardware. Taken together, these examples suggest that in teleoperation-based collection, sensor placement, sensor count, and calibration strategy are not merely hardware choices but core design variables that directly determine data quality and policy performance.

Here, data quality refers not only to the number of collected demonstrations but also to the fidelity and consistency of the sensor-to-action recording process. For VLA-based imitation learning, high-quality data require temporally aligned observation–action pairs, spatially consistent camera and robot calibration, sufficient multi-view coverage to reduce occlusion, reliable tracking of the operator’s motion or command input, and stable recording of contact-related cues when tactile or force feedback is involved. If any of these factors is degraded, the resulting dataset may contain visually rich but causally inconsistent demonstrations, in which the recorded observation does not accurately correspond to the executed action or contact state. Prior studies on data quality in imitation learning and haptic-feedback-based teleoperation have similarly shown that the quality of demonstrations and feedback channels can affect the performance of policies trained from collected data [73,74].

In addition, the quality of teleoperation data also depends on the fidelity of mechanical tracking between operator input and robot motion. If tracking is inaccurate or unstable, demonstration data may accumulate noisy or misaligned observation–action pairs even when the visual recording itself is rich. From this perspective, multi-view or wrist-mounted cameras are useful not only for increasing visual diversity but also for reducing occlusion, whereas consistent calibration and reliable tracking help suppress error propagation caused by calibration noise and motion mismatch.

A closely related issue is the effect of temporal misalignment on causal consistency in imitation learning data. In supervised imitation learning, the policy is trained to predict an action $a_{t}$ from an observation $o_{t}$, which implicitly assumes that the observation and action are correctly paired in time. If the recording pipeline introduces a timestamp offset ∆t between the observation stream and the action log, the dataset may contain spurious pairs such as $\left(o_{t},a_{t+∆t}\right)$, causing the policy to learn actions associated with a state that has already changed. Depending on the end-effector or object velocity, even an offset of 50–100 ms can produce centimeter-level mismatch at the observation–action boundary. In reinforcement learning settings, similar misalignment can corrupt transition tuples $\left(s_{t},{a_{t},\;s}_{t+1}\right)$ and bias value estimation. To mitigate these issues, data collection pipelines should record hardware timestamps at the point of sensor acquisition, align observations and actions before constructing training pairs, and validate temporal consistency by checking whether the recorded action and the subsequent robot state are physically consistent within the intended control interval.

Meanwhile, Open X-Embodiment, or RT-X [17], provides a representative example of integrating multi-institution and multi-robot data into a standardized format, including 22 robots and 527 skills, and demonstrates the possibility of cross robot learning and adaptation beyond dependence on a specific robot or institution. From the perspective of field scalability, studies such as Dobb-E [47], which targets data collection and real-time operation in household environments, indicate a growing trend toward expanding data pipelines without relying on expensive equipment or complex experimental setups.

**Indirect use of human video and web-scale data.** Because robot demonstration data are expensive to collect, large-scale video data of everyday human manipulation provide an attractive alternative for substantially reducing collection cost. R3M [75] showed that a frozen visual encoder learned from human video, including datasets such as Ego4D [76], can improve performance even when only a small amount of robot demonstration data is available and is therefore frequently cited as a representative example of transfer from human video to robotic manipulation. In addition, prior work [77] has shown that robot reward functions can be generalized by measuring task similarity from in-the-wild human videos, thereby suggesting a design space in which the scarcity of robot data can be complemented by human video.

However, this direction is fundamentally constrained by a sensor mismatch problem. Human videos are typically centered on monocular RGB observations and do not provide signals such as tactile feedback, force and torque, or proprioception. As a result, they cannot be directly mapped to the causal state–action relationships required for robotic manipulation. Therefore, the contribution of this category is more accurately understood not as a replacement for action labels, but as a source of representations, rewards, or skill priors.

**Simulation, generation, and augmentation.** Simulation can generate large-scale data at low cost, but it inevitably differs from the real-world in sensor physics, including illumination, material properties, noise characteristics, and the failure patterns of depth sensing. From the perspective of this survey, the key technical issue is therefore not only large-scale data generation but how to reduce the discrepancy between simulated and real sensor behaviors. In practice, this includes mechanisms such as photometric and material variation, sensor-noise injection, modeling of depth failures on transparent or reflective objects, and more explicit treatment of contact-related uncertainty. In this sense, Isaac Gym [78] is important not only because it enables large-scale parallel simulation, but also because it provides a scalable infrastructure for controlled policy training, whereas Isaac Lab [79] further systematizes sensor modeling and task specification so that developers can decide which physical effects should be modeled and to what extent.

Data generation systems such as MimicGen [80] remain valuable, but within the sensor-centered scope of this survey, they are most useful when combined with explicit analysis of which sensor mismatches are being reduced and which failure modes still remain difficult to reproduce. These unresolved gaps include depth failures on transparent or reflective objects, contact noise in tactile and force sensing, and the asynchronous sparse events of event cameras. For this reason, simulation-based results should be interpreted together with real-world validation and failure-mode analysis.

**Comparison of representative VLA training datasets.** Table 4 compares representative datasets for VLA training from the perspective of sensor configuration. Large-scale public datasets are still mostly RGB-centric, and modalities such as haptic sensing and F/T sensing are included in only a portion of limited datasets. Among these, RH20T [81] is an important exception because it incorporates force torque sensing and haptic devices during demonstration collection, thereby addressing contact-rich manipulation more directly. RLBench [82] can also reasonably be included in this category of robot learning datasets and benchmarks. However, unlike fixed real-world datasets such as Open X-Embodiment [17] or DROID [46], RLBench [82] is more appropriately characterized as a large-scale simulation benchmark and data generator designed for training and evaluating VLA models.

A statistical reading of Table 4 reveals three structural patterns in current VLA training data: modality imbalance, scale asymmetry, and task coverage bias. RGB and proprioception are broadly available across the listed datasets, whereas depth is included only in a subset of datasets and may be variable or simulator-dependent in others. Tactile and F/T sensing remain much less common, with explicit F/T coverage appearing mainly in specialized contact-rich datasets such as RH20T. This imbalance implies that policies trained on aggregated datasets may be dominated by visual observations, whereas contact-relevant modalities remain statistically underrepresented. In addition, large-scale datasets such as Open X-Embodiment and DROID provide broad task and environment coverage but do not guarantee uniform tactile or F/T sensing, whereas contact-rich datasets tend to cover narrower task distributions. These patterns suggest that future VLA data collection should report not only dataset scale but also modality coverage, task distribution, sensor noise characteristics, and modality-specific quality-control procedures.

To address this gap, four strategies are recommended. First, existing teleoperation platforms can be retrofitted with wrist-mounted F/T sensors and fingertip tactile sensors (e.g., DIGIT [53] and GelSight [7]) at modest cost. Second, proxy contact signals can be derived post hoc from joint torque estimates or motor currents already present in many existing datasets. Third, simulation platforms such as Isaac Lab [79] and MimicGen [80] natively support contact wrench logging, enabling scalable generation of force-rich demonstrations with domain randomization over contact parameters. Fourth, vision-based force estimation from gripper deformation or optical tactile images [52] can be used to retroactively label existing RGB datasets.

**Mechanistic strategies for reducing simulation–physics discrepancy.** When simulation is used to generate sensor-rich demonstrations, the limitation is not only the visual domain gap but also a simulation–physics discrepancy, which arises when simulated contact, friction, compliance, inertia, actuator response, sensor noise, or timing differ from those in real manipulation. Synthetic demonstrations therefore cannot be assumed to be physically faithful. Several mechanistic strategies can reduce this gap. Physics parameter randomization over mass, friction, restitution, stiffness, damping, actuator delay, and contact-solver parameters prevents the policy from overfitting to a single simulator setting, whereas real-to-sim system identification estimates robot, gripper, object, and contact parameters from real trajectories, force/torque, tactile, and proprioceptive logs to align the simulator with the physical system. Contact-model calibration is especially critical for insertion, sliding, grasping, and deformable- or transparent-object tasks, where small errors in friction or compliance lead to large differences in outcome. Sensor-level discrepancy should also be modeled explicitly by injecting realistic RGB-D noise, depth dropout, tactile saturation, F/T bias, IMU drift, and synchronization delay. Finally, simulation-generated policies should be validated through real-sensor-grounded ablation and perturbation tests rather than simulator success rate alone. In practice, simulation-based data generation is most effective when domain randomization and real-to-sim calibration are combined with residual correction from a small amount of real data and with real-world validation under the deployment sensor configuration.

Together, these strategies provide a practical roadmap for expanding haptic and F/T coverage without requiring entirely new large-scale data collection.

### 3.3. Ground-Truth Annotation for Manipulation

In robotic manipulation, the most critical ground-truth signal is the six-degree-of-freedom (6-DoF) pose of the object and the end effector, represented as a rigid-body transformation T∈SE(3) that jointly encodes rotation R∈SO(3) and translation $t\in R^{3}$ in a single homogeneous matrix. This pose serves not only to guarantee the geometric consistency of observation–action pairs but also to provide a reference for validating the reliability of RGB-D-based three-dimensional measurements discussed in Section 2.1 and proprioception-based pose estimation discussed in Section 2.3. In practice, the acquisition of 6-DoF pose ground truth can be broadly categorized into three approaches: (i) motion capture, (ii) fiducial-marker-based methods, including systems such as AprilTag and ArUco, and (iii) estimation based on depth or RGB-D sensing.

**Motion capture-based methods**. Optical motion capture systems estimate 6-DoF poses at high frequency by tracking reflective markers attached to objects or the robot with multiple infrared cameras. Although accuracy depends strongly on installation quality, calibration, and visibility conditions, motion capture generally provides the highest reference precision in laboratory environments, and performance analyses of Vicon style systems have been reported [84]. However, motion capture has limited scalability for large-scale or distributed data collection across multiple institutions or diverse environments because of (i) high equipment and operational cost, (ii) constraints on installation space and movement paths, and (iii) dropout caused by marker occlusion. Therefore, motion capture is most suitable for small scale benchmarking or validation settings in which highly precise ground truth (GT) is essential, and it often needs to be complemented by other methods when data collection is scaled up.

**Fiducial marker-based methods**. Fiducial markers estimate pose in the camera coordinate frame by detecting two-dimensional markers attached to an object or the environment with an RGB camera. Among these, AprilTag is a widely used representative example, and AprilTag 2 is often cited for improved computational efficiency and robustness [85]. Other marker systems, such as ArUco and ChArUco, can also play similar roles in practical robotic pipelines depending on the required trade-off among robustness, implementation simplicity, and calibration workflow. ArUco markers were originally proposed as configurable square fiducial markers for reliable camera pose estimation and robot localization, whereas ChArUco boards combine ArUco identifiers with chessboard-like corner localization to support accurate camera and hand–eye calibration. Recent VLA-oriented robotic studies have used ArUco or ChArUco markers for camera–robot calibration, simulation-to-real viewpoint consistency, and handheld-camera pose estimation, indicating their practical relevance for pose labeling and spatial grounding in VLA data collection and evaluation [86]. This method can be implemented with a single camera, is relatively low cost, and enables hand–eye validation in both eye-in-hand and eye-to-hand settings, as well as object pose labeling, to be established with comparatively little overhead. However, it has several limitations, including (i) objects to which markers are difficult to attach, such as transparent, small, curved, or irregular objects, (ii) detection failures under illumination changes, motion blur, or partial occlusion, and (iii) the possibility that the marker itself alters the object appearance and creates a gap with the real deployment domain. Accordingly, AprilTag-based ground truth is highly useful as a convenient baseline, but in practice, it is preferable to adopt designs that reduce marker dependence, for example, by using markers only for initialization and then switching to subsequent estimation or tracking.

**Depth- and RGB-D-based methods**. Here, 6-DoF pose estimation from RGB-D point clouds can be applied without markers and is therefore well suited to in-the-wild data collection. Recently, general-purpose 6-DoF pose estimation and tracking models have been proposed. For instance, FoundationPose [87] addresses unified pose estimation and tracking for novel objects. However, the assumptions of this class of methods vary depending on the setting, such as (a) cases that rely on object models including Computer-Aided Design (CAD) or mesh representations and (b) model-free settings in which objects are specified by only a small number of reference images. Therefore, in a paper, it is more accurate to explicitly state the required conditions than to make categorical claims such as CAD is entirely unnecessary. BundleSDF [88] proposes a framework that jointly performs tracking and three-dimensional reconstruction of unknown objects from RGB-D sequences and can be regarded as one useful direction for generating or refining GT when no prior CAD model is available. At the same time, depth-based approaches inherit the physical limitations of the sensor itself. For transparent or highly reflective objects, depth measurement often fails or becomes severely noisy, which directly reduces the reliability of pose ground truth. The main advantage of depth-based ground truth lies in its scalability; however, failure modes associated with object type and surface properties should be explicitly stated, together with quality control procedures such as filtering, uncertainty tagging, and relabeling.

In practical implementations, hybrid pipelines are frequently adopted. A representative strategy is to first obtain a stable initial pose with AprilTag for initialization or validation and then continuously update it through RGB-D-based estimation or tracking. Another common approach is to secure an accuracy reference baseline with small-scale motion capture and then expand to large-scale data collection with RGB-D methods. Such designs show that modality-specific failure modes (Section 2) can also become bottlenecks at the ground-truth stage and, therefore, that ground-truth reliability itself cannot be separated from sensor design and selection.

## 4. Multi-Modal Fusion Architectures in VLA

This section examines how heterogeneous sensor signals are transformed into policy-compatible representations, fused within VLA architectures, and linked to robot action generation. Multi-modal VLA models generally do not operate directly on raw sensor streams; each modality is first encoded through an appropriate tokenizer, encoder, or adapter that reflects its structure, sampling characteristics, and physical meaning. The resulting representations are then integrated through fusion mechanisms such as early fusion, late fusion, cross-attention, token-level fusion, adapters, or mixture of experts.

In this survey, the “fusion design space” denotes the set of architectural choices that determine how heterogeneous sensor signals are encoded, temporally aligned, and integrated within a VLA policy. This space is organized along four axes: fusion timing, modality interaction depth, missing-modality robustness, and computational cost. Fusion timing refers to whether modalities are combined at the input, intermediate, or output stage; modality interaction depth indicates how explicitly cross-modal correlations are modeled; missing-modality robustness describes how the system behaves when one or more sensor streams are unavailable or degraded; and computational cost captures the latency and memory overhead introduced by the fusion module.

From this perspective, multi-modal fusion in VLA should be understood not only as a representation-learning problem but also as a structured design problem that connects sensing, internal reasoning, and action generation.

### 4.1. Fusion Strategies for Heterogeneous Sensors

In multi-modal VLA, fusion is not merely the declaration that multiple sensors are used together. Rather, it refers to a structural design choice that transforms signals with different measurement principles, noise characteristics, and sampling rates into conditioning information that can be effectively exploited by the policy. Accordingly, fusion strategies should not be evaluated through a single ranking of which method is better. Their suitability instead depends on (i) the degree of sensor heterogeneity, (ii) the requirements imposed by latency and synchronization, (iii) the likelihood of modality missingness, and (iv) the resolution of cross-modal interaction required, ranging from low-level physical correlation to high-level semantic alignment. For this reason, fusion performance depends not only on representation design but also on whether heterogeneous sensor streams are temporally aligned in a causally consistent manner. In this section, the design space is organized into three categories: early fusion, late fusion, and hybrid fusion.

From an information-theoretic perspective, the marginal value of modality *m* can be expressed as the conditional information gain $I\left(a_{t};r_{t}^{\left(m\right)}|r_{t}^{\left(-m\right)}\right)$, where $a_{t}$ denotes the action to be predicted, $r_{t}^{\left(m\right)}$ denotes the representation of modality *m*, and $r_{t}^{\left(-m\right)}$ denotes the representations of the remaining modalities. This quantity represents the information contributed by modality *m* beyond what is already available from the other sensors. This formulation explains why tactile and F/T signals can remain informative in contact-rich tasks even when visual observations appear sufficient. It also provides a formal criterion for interpreting modality contribution beyond benchmark success rate alone.

A complementary way to formalize multi-modal fusion is through Bayesian state estimation. Let $x_{t}$ denote the latent task-relevant state and $z_{t}^{\left(m\right)}$ the observation from modality *m*. With a measurement model $z_{t}^{\left(m\right)}=h_{m}\left(x_{t}\right)+\epsilon_{t}^{\left(m\right)}$, the fused belief can be written as

(2) $$p(x_{t}|z_{t}^{\left(1\right)},\ldots ,z_{t}^{\left(M\right)})\propto p(x_{t})\prod_{m=1}^{M}p(z_{t}^{\left(m\right)}|x_{t})$$

under the conditional-independence assumption. This formulation makes explicit that each modality contributes a likelihood term with its own noise characteristics. IMU and proprioceptive signals are often modeled with Gaussian noise and bias drift, whereas vision, depth, and tactile observations may exhibit non-Gaussian, outlier-prone, or heavy-tailed uncertainty. Therefore, practical fusion may require Kalman filtering, particle filtering, robust estimation, or factor-graph optimization depending on the modality and task. This Bayesian view also highlights the importance of observability, sensor correlation, and uncertainty propagation from perception to action.

**Interaction between sensor configuration and learning objectives.** Since fusion architectures are ultimately trained through policy-learning objectives, sensor configuration also affects the optimization problem. Learning-based VLA policies are trained through objective functions such as imitation learning losses, diffusion or flow-matching losses, and multi-objective losses. For imitation learning, a typical formulation is

(3) $$L_{BC}=E_{\left(o_{t},a_{t}\right)}[l\left(a_{t},\pi_{\theta }\left(o_{t}\right)\right)]$$

where $o_{t}$ is the multi-modal observation, and *ℓ*(⋅) denotes a task-dependent action prediction loss. In multi-modal policies, this task loss may be combined with auxiliary terms,

(4) $$L=L_{task}+{\lambda_{align}L}_{align}+{\lambda_{rec}L}_{rec}+{\lambda_{safe}L}_{safe}$$

where the additional terms encourage cross-modal alignment, modality-specific reconstruction, or safety-aware behavior. Adding sensor modalities can improve observability and action prediction, but it also increases input dimensionality, sample complexity, and the risk of modality imbalance. Therefore, sensor-rich VLA training should consider not only architecture design but also loss balancing, gradient conditioning, and the possibility that sparse modalities such as tactile or F/T signals may be underutilized when RGB dominates the training objective.

**Early fusion.** This approach constructs a shared token sequence from different modalities at the input stage, or at a very shallow layer, so that a single encoder or policy network learns a joint representation from the outset. For modality combinations such as language and RGB, where internet-scale pre-training infrastructure is already rich, early fusion can achieve semantic alignment relatively directly and is simple to implement. However, when signals with substantially different forms and statistics, such as tactile images, force and torque time series, and proprioceptive vectors, are flattened and mixed under the same tokenization convention, modality specific physical properties may be lost, including the local patterns of contact and slip or the spikes and delays in force signals, and the domain gap may even be amplified. To reduce the loss of modality-specific physical information, a practical strategy is to process each sensor type through a dedicated branch before fusion. For example, multi-branch convolutional architectures can assign separate branches to different data types, allowing each branch to preserve the native structure of its input, such as spatial appearance in RGB or depth images, local deformation patterns in tactile images, and temporal variations in force/torque or proprioceptive signals [89]. The resulting modality-specific features can then be fused at an intermediate or high-level representation space through concatenation, attention, gating, or cross-attention. The effectiveness of integrating complementary feature types, such as spatial appearance, frequency-domain signatures, and optical flow, has also been demonstrated in AIGC video detection. This suggests that heterogeneous feature fusion can serve as a broadly applicable design principle for modeling complementary appearance and motion cues [90].

This design is particularly useful when the physical meaning of each modality should be retained before cross-modal interaction, and it provides a concrete architectural alternative to naive early fusion. In addition, as the number of modalities increases, the sequence length also grows, which introduces a scalability limitation in self-attention-based policies because computation and memory costs increase rapidly.

**Late fusion.** This approach processes each modality independently through a dedicated encoder or tokenizer and then combines them in a high-level representation space. For example, Octo [15] separates observation tokenizers by modality and embeds RGB, language, and proprioceptive states independently before integrating them in the policy network, thereby presenting a general framework in which heterogeneous sensors can be added or replaced incrementally. Efficient temporal feature aggregation is also important for real-time visual perception in robotic systems. For example, adaptive sparse memory mechanisms in video object segmentation can reduce redundant computation across sequential frames while preserving robust segmentation performance [91]. The practical advantages of late fusion are twofold. First, preprocessing, normalization, and missing modality handling can be encapsulated at the level of each module. Second, structural robustness can be more easily achieved because a sensor-specific module can simply be deactivated when that modality is missing or of degraded quality. However, in situations where low-level cross-modal correlations are important, such as spatial correspondences between tactile and visual signals or the relationship between force signals and visually observed contact events, a structure in which modalities are processed entirely independently may limit the learning of fine-grained interactions.

**Hybrid fusion.** This approach preserves modality-specific encoders while learning interactions across modalities through cross-attention or constrained interaction modules, thereby striking a balance between the representational power of early fusion and the modularity of late fusion. A representative example is Flamingo [92], which introduced the Perceiver Resampler and gated cross-attention to connect frozen vision and language backbones, enabling flexible multi-modal integration even when text and image, or video, inputs are arbitrarily interleaved. BLIP-2 [93] does not directly connect the image encoder to the large language model but instead introduces an intermediate module called Q-Former to transfer visual information to the language side. Unlike a simple early fusion strategy that mixes all tokens into a single pool, this design can be interpreted as a more structured approach that simultaneously targets computational efficiency and stable alignment by explicitly controlling the entry point and form of visual information. PaLM-E [33] presents another hybrid path by projecting continuous sensor inputs, such as joint states and poses, into the language embedding space and coupling them with an LLM, thereby aligning physical signals with the internal representations used for language-conditioned action generation. More recently, GR-2 [94] has shown a further direction by first acquiring representations of world dynamics through web-scale video pre-training and then extending this into a generative VLA framework that jointly handles video generation and action prediction using robot trajectories, thereby integrating video, language, and action signals within a single model.

**Computational and representational trade-offs of fusion strategies.** The three fusion strategies differ not only in where modalities are integrated but also in computational complexity, memory usage, and the degree of cross-modal interaction they support, as shown in Table 5. Let $L_{m}$ denote the number of tokens from modality m, $L=\sum_{m}L_{m}$ the total token length, *M* the number of modalities, and *d* the token dimension. In early fusion, all tokens are concatenated and processed by a shared self-attention backbone, leading to an attention cost of $O(L^{2}d)$ and memory cost of $O(L^{2})$ per layer. This provides dense token-level interaction across modalities but becomes expensive as additional sensors are introduced. In late fusion, each modality is first encoded independently and then fused through concatenation, gating, or a lightweight MLP. The fusion-stage cost can be reduced to approximately O(Md) after modality-level summarization, but this efficiency comes at the cost of losing low-level cross-modal correlations before fusion. From an information-theoretic perspective, if $z_{t}^{\left(m\right)}=\emptyset_{m}(x_{t}^{\left(m\right)})$ is a compressed representation of modality mmm, then information relevant to action prediction may be discarded unless $z_{t}^{\left(m\right)}$ is sufficient for the downstream policy. This is particularly problematic in contact-rich manipulation, where the relationship between visual contact cues and F/T or tactile signals may only be recoverable through joint or intermediate interaction. Hybrid fusion addresses this trade-off by preserving modality-specific encoders while introducing cross-attention between selected modality pairs, with a typical cost of $O(L_{q}L_{kv}d)$ per cross-attention operation. Thus, hybrid designs provide more structured cross-modal interaction than late fusion while avoiding the full quadratic cost of unconstrained early fusion when sparse or hierarchical attention is used.

Beyond this taxonomy, graph-based and probabilistic fusion provide useful complementary perspectives. In graph-based fusion, modalities or sensor-specific features are represented as nodes, and edges encode spatial, temporal, or semantic relationships among sensors. This structure is useful when the physical sensor topology is sparse or known in advance, such as the geometric coupling between a wrist camera and a wrist-mounted F/T sensor. In probabilistic fusion, each modality contributes uncertain evidence about a latent task state, and the fused estimate is represented as a posterior belief through Kalman filtering, particle filtering, or factor-graph inference. Although such probabilistic methods may be costly for direct VLA inference, they are valuable for calibration, state estimation, uncertainty-aware fusion, and reliability weighting in deployment-oriented systems.

The choice of fusion strategy is not simply a matter of how early the modalities should be mixed but rather a design problem that must simultaneously satisfy the requirements imposed by sensor heterogeneity, modality missingness, latency, and the desired resolution of cross-modal interaction. In particular, as physically heterogeneous signals such as tactile sensing, F/T sensing, and event cameras are incorporated alongside RGB, late or hybrid fusion often becomes a more reasonable default from the standpoint of real-world deployment because it preserves modality-specific processing while allowing interaction only at the points where it is actually needed.

### 4.2. Reliability-Aware and Robust Fusion

In real-world robotic manipulation, the assumption that sensors are always operating normally rarely holds. Camera quality can deteriorate sharply due to illumination changes, backlighting, motion blur, or contamination. Depth sensing frequently suffers from missing measurements on transparent or reflective objects, and tactile signals can be distorted by poor contact or sensor wear. Accordingly, it is not sufficient to merely integrate multiple modalities. The real deployment reliability of VLA critically depends on an explicit reliability-weighting mechanism that can automatically shift trust toward other modalities when a particular sensor fails or degrades.

The broader multi-modal learning literature has repeatedly shown that fusion schemes are vulnerable to modality missingness and degradation. Ma et al. [95] systematically demonstrated that multi-modal Transformers can suffer substantial performance drops when some input modalities are partially missing, and similar vulnerabilities to sensor inconsistency have also been noted from a robotics perspective [96]. This suggests that what matters is not only average performance improvement but also context-aware fusion that can determine which modality should be trusted more under a given condition.

However, at the current stage of VLA research, examples that adopt modality reliability reweighting as a standard design principle remain very limited. Generalist policies such as Octo [15] separate modality-specific tokenizers to improve scalability and learn inter-token relations through Transformer self-attention, but this is closer to implicit weighting learned from data. In other words, most existing systems still lack a reliability-aware module that can diagnose, in real-time, whether the RGB input in the current frame is degraded or the depth signal is corrupted and then actively shift contribution toward tactile or proprioceptive tokens that are physically more reliable under the given condition [11,15]. This architectural gap constitutes a clear research problem because sensor degradation may otherwise leave the model behavior ill-defined or even lead to unsafe actions.

There are three implementation paths that may help address this gap. The first is uncertainty-based weighting. This approach estimates modal prediction uncertainty, including stochastic and perceptual uncertainty, and reduces the contribution of low-confidence channels. Recent evidential learning approaches point to a promising direction for suppressing the influence of highly noisy channels [97]. The second is gating-based dynamic fusion. This method dynamically rescales the contribution of each modality according to the input state and can maintain stability by redistributing weights even under missing-modality conditions [98]. The third is robust fusion based on quality cues. This approach directly adjusts attention weights using cues such as visual sharpness or tactile contact quality [99]. In manipulation-oriented VLA, this can provide a concrete design basis for implementing stage-dependent reliability transfer, in which the policy relies primarily on vision before contact and shifts toward physical feedback after contact.

The three weighting strategies differ substantially in computational overhead. Quality-cue-based methods—which derive weights from simple proxy signals such as image sharpness or tactile contact presence—add negligible latency and are therefore compatible with high-frequency control loops [99]. Gating-based modules impose moderate overhead comparable to a small MLP and can run in parallel with the main inference [98]. Uncertainty-based approaches such as evidential learning [97] carry the highest cost; in latency-sensitive tasks, their overhead can be bounded by running estimation asynchronously so that the policy consumes the most recently computed weights without stalling the control loop [100,101]. In high-speed manipulation where cycle times are tight, quality-cue or gating-based modules are therefore the more practical default, whereas uncertainty-based weighting is better suited to lower-frequency planning stages.

### 4.3. Action Representation and Temporal Alignment with Sensor Resolution

Beyond input-side fusion, the executability of a VLA system also depends on the form of action representation and the frequency at which actions are updated. Therefore, this section analyzes how action chunking and closed-loop stepwise or streaming execution interact with sensor update rates.

**Action chunking and sensor update rates**. Action chunking is an open-loop strategy in which multiple future actions are predicted and executed at once from a single observation, and it has been widely adopted in ACT [59] and diffusion policy-based methods [13]. ACT uses action chunk prediction together with temporal ensembling, whereas diffusion policy generates an action sequence over a finite horizon and performs control in a receding-horizon manner. Such architectures offer clear advantages in reducing the number of inference calls and stabilizing action generation. However, when the chunk length is long or the replanning frequency is low, newly arriving high-frequency sensor information cannot be immediately reflected in action generation during chunk execution. This limitation is particularly critical for events that occur on short time scales, such as contact changes, slip, or unexpected external forces, and it may substantially restrict the effective use of F/T sensors, tactile sensors, and high-frequency proprioceptive signals.

**Closed-loop stepwise/autoregressive and streaming execution**. In this survey, the term autoregressive execution is used in the context of robot policy execution, not in the sense of autoregressive system identification or time-series modeling. This usage differs from autoregressive modeling in data-driven system identification. In system identification, autoregressive or autoregressive-with-exogenous-input (ARX) models typically aim to estimate an explicit input–output dynamic model, in which the current or future output is represented as a function of previous outputs, previous inputs, and noise terms. The main objective is therefore to identify model structure, terms, or coefficients that explain the dynamics of the target system [102]. By contrast, autoregressive execution in VLA-based manipulation does not aim to recover an explicit parametric dynamic model of the robot or environment. Instead, it refers to a closed-loop stepwise action-generation process in which the policy produces or updates the next executable action conditioned on the latest observation, the current robot state, and, when applicable, previously generated actions or an action buffer. Therefore, the key distinction from action chunking is that newly arriving sensor feedback can be incorporated during execution rather than waiting until the end of a fixed open-loop action chunk.

Recent streaming-style policies can implement this idea by maintaining a future-action buffer, reusing part of the previously generated sequence, and updating the immediately executable action based on newly available observations. For example, SFP [61] implements a closed-loop structure that conditions action generation on the latest observation at every sampling step through a reparameterization that linearly shifts the time step according to the action index. SDP [60] rolls over the partially denoised sequence from the previous step and uses it as the initialization for the next inference step, thereby reducing inference cost while maintaining a tighter sensor–action loop. This line of work suggests that, even without abandoning action chunking altogether, shorter replanning intervals and partial refresh can substantially improve the utilization of high-frequency feedback.

**The multi-rate gap between sensors and VLM inference**. Sensors with very high temporal resolution, such as IMUs and event cameras, are typically not directly matched to the relatively low-frequency inference cycle of VLM or VLA systems [39]. Three representative approaches have been proposed to address this gap. First, hierarchical control decomposition [100] allows a high-level policy to generate target poses or sub-goals at a low frequency, whereas a low-level controller tracks them using high-frequency sensor feedback. Second, summary-feature-based integration aggregates high-frequency signals over a temporal window into statistics such as mean, variance, peak value, or event occurrence and uses these summaries as inputs to the low-frequency policy. Event cameras can likewise be incorporated by converting asynchronous events into accumulated representations such as event frames or surfaces of active events [39]. Third, asynchronous event triggering maintains low-frequency inference during normal operation but immediately invokes replanning only when specific events, such as slip or impact, are detected.

Such multi-rate designs provide a practical means of connecting the responsiveness of high-frequency sensors to actual policy execution without substantially increasing computational cost [101]. As a concrete guideline, when a 1 kHz IMU or F/T stream is paired with a VLA backbone running at 1–10 Hz, the following re-planning cadences are recommended: (i) for hierarchical decomposition [100], the high-level policy re-plans at 1–5 Hz whereas the low-level controller tracks at the full sensor rate; (ii) for summary-feature integration, features are aggregated over a 100–200 ms window and fed to the backbone at each inference step; and (iii) for asynchronous event triggering, baseline re-planning is kept at 1–5 Hz, with immediate replanning invoked upon detecting contact events such as slip or impact threshold crossings. These ranges are intended as practical starting points; the appropriate cadence should be validated empirically for each platform and task, as the optimal frequency depends on the specific robot kinematics and the tightness of the required control loop.

This cadence selection is closely related to the latency-induced delay between sensing, fusion, and action generation. From a control perspective, perception and fusion latency can be modeled as a delay τ, such that the policy computes $a_{t}=\pi (o_{t-\tau })$. In contact-rich or high-speed manipulation, where slip onset, impact, or force transients may occur within milliseconds, τ should be kept below the characteristic time scale of the relevant physical event. Latency should therefore be reported not only as an efficiency metric but also as a stability- and safety-relevant criterion.

A practical way to reason about this issue is through the concept of a latency budget. In a typical VLA deployment, the total loop latency consists of three major components: sensor acquisition and preprocessing, multi-modal fusion and policy inference, and action transmission with actuator execution. This can be written as $\tau_{total}=\tau_{sense}+\tau_{infer}+\tau_{act}$. When $\tau_{total}$ approaches or exceeds the characteristic time scale of task-relevant physical events, such as slip onset or impact transients, the outer VLA policy may no longer react quickly enough to preserve stable contact. These event time scales are task and hardware dependent, but slip or impact-related changes can often occur on the order of milliseconds to tens of milliseconds. Therefore, the VLA policy loop and the inner actuator control loop should be co-designed rather than treated independently. In practice, the inner loop, typically running at hundreds of Hz to kHz in impedance- or torque-controlled robots, should handle high-frequency force regulation and rapid disturbance rejection, whereas the outer VLA loop updates goals, task context, and compliant behavior at a lower rate. If sensing latency or inference cost makes the outer-loop response slower than the relevant contact dynamics, the system should rely on low-level compliance, reflexive control, predictive buffering, streaming execution, or short-horizon replanning to decouple fast actuation from slower multi-modal inference.

**Figure 4:**
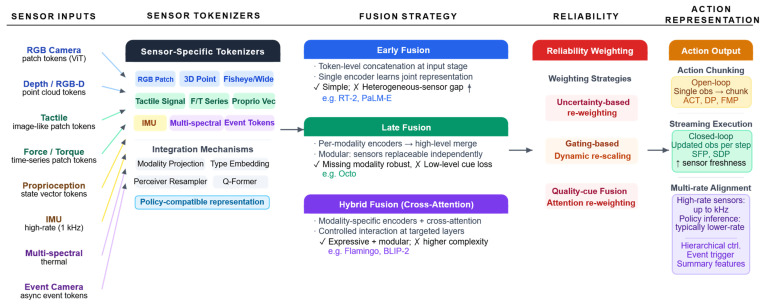
Multi-modal fusion and action-representation pipeline in VLA. The pipeline illustrates three design components: fusion strategies, modality reliability weighting, and action representation with temporal alignment. SFP = Shifted Flow Policy, SDP = Streaming Diffusion Policy, DP = Diffusion Policy, FMP = Flow Matching Policy, and ACT = Action Chunking with Transformers.

Figure 4 illustrates the overall multi-modal fusion pipeline, including three major fusion strategies, modality reliability weighting mechanisms, and the action representation process under temporal alignment considerations.

## 5. Benchmarks and Evaluation Protocols

Section 3.2 and Table 4 primarily summarize how VLA training data are collected and what sensor configurations they include. In contrast, the focus of this section is to examine which sensor configurations are actually evaluated by current representative benchmarks and evaluation protocols.

### 5.1. Limitations of Sensor Coverage in Existing Benchmarks

Recent VLA research has expanded rapidly in terms of task diversity, robot diversity, and language diversity, yet its evaluation frameworks remain relatively limited in terms of sensor diversity. For example, downstream evaluations based on Open X-Embodiment [17] cover a wide range of robotic platforms, but the observation setup is still often centered on camera-based inputs and end-effector action representations. Octo demonstrated the potential of a generalist policy built on such large-scale data. However, the associated benchmark settings and downstream evaluations are likewise often structured around camera-based observations and end-effector action representations. In other words, although the diversity of robots and tasks has increased substantially, sensors such as tactile sensing, F/T sensing, and IMUs have not yet been systematically incorporated as central axes of benchmark design.

Similar patterns can be observed in other widely used benchmarks as well. LIBERO [103] is a representative benchmark for evaluating continual learning and transfer in language-conditioned manipulation across different knowledge components, including spatial relations, objects, goals, and scenes. BEHAVIOR [104,105] addresses long-horizon tasks in more realistic household environments. VLABench [106] proposes a manipulation benchmark aimed at separately evaluating language understanding and physical reasoning ability. However, these benchmarks also rely primarily on RGB or RGB-D centered observation structures and do not treat comparison across sensor configurations as a primary evaluation target. As a result, current benchmarks are useful for assessing visual grounding and the execution of high-level instructions but remain limited in their ability to directly evaluate contact-based manipulation or the effective use of high-frequency feedback.

This limitation becomes particularly pronounced in contact-rich manipulation. In tasks such as insertion, threading, cable arrangement, deformable object manipulation, and fine assembly, success cannot be determined solely from visual information. In reality, signals such as force changes, slip, instantaneous impacts, and pose fluctuations play a critical role. However, current benchmarks often fail to isolate and reveal the contributions of these sensor channels. Consequently, even when two policies achieve the same success rate, it is difficult to distinguish a case in which one policy relies on unstable visual heuristics from another in which the policy operates more robustly by exploiting contact information.

Accordingly, future VLA benchmarks must move beyond purely task-centric design and explicitly incorporate sensor-centric design. More specifically, at least the following four elements should be defined jointly:

- First, the set of observations provided to the policy should be specified, including modalities such as RGB, RGB-D, wrist cameras, tactile sensing, F/T sensing, and IMUs.
- Second, the sampling rate of each sensor and the synchronization protocol among sensors should be clearly defined.
- Third, the benchmark should state whether it is intended to evaluate primarily vision-centric problems, contact-centric problems, or robustness under perturbation and recovery.
- Fourth, the evaluation design should include mechanisms such as ablation studies or sensor dropout conditions that make it possible to quantitatively isolate and interpret the contribution of each sensor channel.

Only with such design principles can future benchmarks evaluate not merely whether a policy succeeds, but also which sensor configuration enables success under which conditions.

### 5.2. Limitations of Current Evaluation Metrics and a Proposal for a Multidimensional Evaluation Framework

In current robotic manipulation and VLA research, success rate (SR) is one of the most commonly adopted default evaluation metrics in representative benchmarks. This is mainly because the SR is intuitive, easy to compute, and allows for direct comparison across methods and tasks. However, this statement should be understood as a qualitative observation based on representative benchmarks rather than as a bibliometric occurrence analysis across the entire literature. Because the SR is intuitive and easy to compare across methods, it is employed as the default metric in many benchmarks, including LIBERO [85], BEHAVIOR [104,105], and VLABench [106]. However, as sensor configurations become more diverse and as long-horizon and contact-rich tasks become more prevalent, it becomes increasingly difficult for a single success-rate metric to adequately characterize the actual performance of a system. In particular, the SR cannot distinguish between a policy that barely completes a task and a policy that performs the same task in a stable, repeatable, and well-controlled manner. Furthermore, it does not adequately reflect safety, execution quality, replanning costs, and inference efficiency during task execution.

In general, SR is defined as follows:

(5) $$SR=\frac{1}{N}\sum_{i=1}^{N}1({success}_{i})\times 100$$

where *N* denotes the total number of evaluation episodes and equals 1 if the i-th episode is successful and 0 otherwise. The main advantage of this metric lies in its simplicity and interpretability. However, its limitation is that it cannot capture the quality of success. For example, one policy may succeed only after multiple corrective motions and excessive contact, whereas another may complete the same task in a smoother, safer, and more consistent manner. Nevertheless, if both policies are ultimately successful, they will receive the same SR. In other words, the SR indicates only whether the work has been completed, not how well it was completed. This limitation has also been pointed out by Li et al. [107] and Liu et al. [108], and there is growing recognition that final task completion alone is insufficient for assessing the true deployability of a policy.

A second limitation is that the SR does not adequately reveal sensor robustness. In practice, it should be measured separately which policy maintains performance more stably under perturbations such as camera noise, illumination changes, sensor latency, calibration error, tactile dropout, or F/T spikes. To address this issue, the following robust success rate (RSR), can be defined:

(6) $$RSR=\frac{1}{\left|P\right|}\sum_{p=1}^{P}SP(p)$$

where *P* denotes the set of perturbation conditions, and SP(p) is the success rate under a specific perturbation condition *p*. This metric is useful for measuring average robustness. However, an average value alone may fail to reveal cases in which performance collapses under a particular perturbation, that is, edge-case failures. Therefore, from the perspective of real deployment, it is desirable to report the worst-case success rate (WSR), together with RSR.

(7) $$WSR={min}_{p\in P}SR(p)$$

This metric represents the lower bound of policy performance under the worst perturbation condition in the given perturbation set. For example, even if a policy achieves a high average robust success rate, the WSR can immediately expose its vulnerability if the policy almost always fails under a particular illumination condition or a specific sensor-dropout setting. Therefore, reporting both the robust SR and WSR makes it possible to evaluate, at the same time, the average stability of a policy and the boundary of its failure cases.

A third limitation is that efficiency is not reflected. In real systems, it is important to assess not only whether a policy succeeds but also how many replanning steps it requires, what its average inference time is, and how much computational resource is consumed per successful execution. For example, average inference latency (AIL) can be measured as follows:

(8) $$AIL=\frac{1}{N}\sum_{i=1}^{N}T_{i}$$

Here, $T_{i}$ denotes the inference time for the *i*-th episode or action step. In addition, metrics such as Cost per Success (CpS), are also practically useful.

(9) $$CpS=\frac{Total\;Compute\;Cost}{Number\;of\;Successful\;Episodes}$$

This metric is necessary for assessing whether a high performing policy remains within a reasonable resource budget in actual deployment. In particular, for large-scale VLM or VLA models, even if the success rate is slightly higher, a policy is unlikely to be preferred in practice if it incurs excessive inference latency or replanning cost.

The occurrence column in Table 6 indicates the qualitative reporting tendency observed across representative VLA and robotic manipulation studies, rather than a formal percentage-based frequency count. A strict metric-frequency analysis would require a separate systematic coding protocol, whereas the purpose of this table is to provide a practical evaluation guide for sensor-rich VLA systems.

A fourth limitation is that safety and execution quality are not sufficiently reflected in current benchmarks. This issue is especially important in contact-rich manipulation, where quantities such as peak force, collision count, slip count, trajectory jerk, and corrective action frequency become critical. These factors should be reported separately from success rate and serve as key evidence for determining whether a given sensor configuration actually contributes to safe manipulation. As a recent effort to address this issue more directly, Liu et al. [108] emphasized the need for a trustworthy evaluation framework that includes execution quality, smoothness, and safety.

To address these limitations, this survey proposes a multidimensional evaluation framework that organizes VLA evaluation along four axes:

- Task Completion ($S_{task})$: SR, sub-goal completion rate, and partial progress score;
- Robustness ($S_{robust})$: RSR, WSR, sensor dropout sensitivity, and recovery after perturbation;
- Safety and Execution Quality ($S_{safe}$): peak force, collision count, slip count, smoothness, and jerk;
- Efficiency ($S_{eff}$): inference latency, control frequency, replanning count, and energy or compute cost.

To clarify the proposed multidimensional evaluation perspective without turning this survey into a bibliometric metric-frequency study, Table 6 provides a compact summary of major evaluation axes for sensor-rich VLA systems. The occurrence column is reported qualitatively because existing benchmarks differ substantially in task definition, sensor configuration, and reporting protocol.

As summarized in Table 6, SR is commonly reported as a primary task-completion metric because of its simplicity, intuitive interpretation, and ease of comparison across methods. The occurrence labels in Table 6 should therefore be interpreted as qualitative indicators of reporting practice rather than as percentage-based results from a formal bibliometric metric-frequency analysis. However, deployment-oriented VLA systems require complementary metrics that separately reveal robustness, safety, real-time feasibility, and the contribution of individual sensor modalities.

To further operationalize these evaluation axes, three additional metric definitions can be used. First, robustness can be quantified through perturbation sensitivity:

(10) $$\rho \left(\delta \right)=\frac{{SR}_{clean}-SR(\delta )}{\delta }$$

where ${SR}_{clean}$ is the success rate under unperturbed conditions, and SR(δ) is the success rate under a perturbation of magnitude δ, such as sensor noise, calibration error, frame dropout, or tactile/F/T signal corruption. A lower ρ(δ) indicates that the policy is less sensitive to sensor perturbation. Second, safety can be measured as a normalized event-penalty score:

(11) $$S_{safe}=1-\frac{\alpha N_{col}+\beta N_{force}+\gamma \;N_{slip}}{N_{max}}$$

where $N_{col}$, $N_{force}$, and $N_{slip}$ denote the number of collisions, force-threshold violations, and slip events, respectively. The weights *α*, *β*, and *γ* reflect task-dependent failure severity, and $N_{max}$ is a normalization constant. Third, end-to-end policy-loop latency can be decomposed as

(12) $$\tau_{total}=\tau_{sense}+\tau_{fuse}+\tau_{infer}$$

where $\tau_{sense}$ denotes sensor acquisition and preprocessing time, $\tau_{fuse}$ denotes multi-modal fusion time, and $\tau_{infer}$ denotes policy inference time. Reporting these components separately helps identify whether the bottleneck arises from sensor I/O, fusion architecture, or model inference.

If needed, these metrics can be normalized and summarized into a single composite score. An example is given as follows:

(13) $$Overall\;Score=w_{1}\cdot S_{task}+w_{2}\cdot S_{robust}+w_{3}\cdot S_{safe}+w_{4}\cdot S_{eff}$$

where $S_{task}$, $S_{robust}$, $S_{safe}$, and $S_{eff}$ denote normalized scores in the range from 0 to 1, and $w_{1}+w_{2}+w_{3}+w_{4}=1$. Appropriate weight values are inherently domain dependent and should therefore be interpreted in relation to the target application. For instance, task completion may be prioritized in general manipulation benchmarks, whereas safety may deserve greater emphasis in contact-rich or deployment-critical scenarios. As no universal weight assignment can be justified without empirical validation, including domain-specific ablation studies or failure-cost analyses, this study refrains from prescribing fixed values. Instead, this paper recommends that future studies explicitly report the chosen weights together with the underlying rationale, thereby supporting transparent and reproducible cross-paper comparisons.

However, in actual benchmarks, it is generally more desirable to present each axis as a separate performance profile rather than relying solely on a single aggregate score. Although a unified score simplifies comparison, it can obscure what a given policy is strong at and where it remains weak. In fact, BEHAVIOR [104,105] has already suggested moving beyond binary success by incorporating task progress and efficiency, and Liu et al. [108] more directly emphasized the need for quality-aware evaluation.

From a practical standpoint, it is recommended that each paper and benchmark report at least the four metrics defined in Equations (5)–(8). Once such a protocol is adopted, future VLA research will be able to move beyond the simple question of whether a policy succeeded and instead compare how robustly, how safely, and how efficiently a given sensor configuration enabled success. This is particularly essential for quantitatively revealing the value of modalities such as tactile sensing, F/T sensing, IMUs, thermal, and event-based vision, all of which are especially important in real-world deployment settings. This reporting practice also provides the basis for deciding whether an additional sensing source is worth its computational cost, as discussed in Section 2.2. To this end, the tolerability of an added modality should be evaluated not by latency alone but by the joint profile of performance gain and overhead: sensor-rich VLA studies should report both the improvement obtained by adding a modality, such as the change in success rate, the reduction in slip or collision events, or the robustness gain under perturbation, and the corresponding overhead, such as the increase in average inference latency, the increase in policy-loop latency, memory usage, and compute cost. Reporting both terms makes it possible to determine whether a sensing source is merely more informative or actually beneficial under the latency and safety constraints of the target task in the sense formalized in Section 2.2.

## 6. Open Problems and Future Directions

The preceding sections have reviewed the sensor modalities used in VLA systems, data collection pipelines, multi-modal fusion structures, action representations, and the limitations of current benchmarks and evaluation metrics. Taken together, these discussions suggest that the central challenge for future VLA research is not simply to design larger language models or stronger action decoders. Rather, the next stage of progress depends on how heterogeneous physical sensors can be integrated into generalizable representations, how such representations can be aligned with actual control loops, and how robustness and safety can be ensured under real deployment conditions. From this perspective, this section outlines five directions that should receive particular attention in future research.

### 6.1. Sensor-Agnostic VLA Architectures

Most current VLA systems are still designed primarily around RGB or RGB-D inputs, whereas sensors such as tactile sensing, force/torque, IMU, thermal sensing, and event cameras are incorporated only selectively. As a result, whenever the available sensor configuration changes across platforms or environments, the input encoders, normalization schemes, and fusion strategies often need to be redesigned. In the long-term, sensor-agnostic VLA architectures are needed that can adapt flexibly even when the type and number of sensors vary.

This problem cannot be solved simply by increasing the number of input channels. This is because sampling rates, noise characteristics, spatial resolution, and semantic density vary significantly from sensor to sensor. Accordingly, future research should consider, in an integrated manner, standardized token interfaces that can map heterogeneous sensors into a shared latent space, adaptive fusion mechanisms that minimize performance degradation under missing-modality conditions, and modular architectures that can be extended to new sensors without retraining the entire backbone [15,17]. Ultimately, the key evaluation criterion for VLA architectures should shift from which sensors are used to how effectively the policy adapts when the sensor configuration changes.

### 6.2. Tactile and Force Grounding in Language-Conditioned Manipulation

Although the integration of vision and language has already become a mainstream direction in VLA research, the problem of connecting tactile and force signals with language remains largely underexplored [54,109,110]. Yet many concepts that are important in real manipulation are more directly revealed through contact than through vision. For example, instructions such as “push it gently,” “stop when resistance is felt,” or “grasp it firmly but do not crush it” are difficult to ground adequately through vision alone and instead require coupling with tactile and force signals.

This direction involves two central challenges. First, tactile and force signals must be represented not merely as auxiliary inputs but as semantically meaningful units that can be interpreted in linguistic terms. Recent work such as UniTouch [109] has demonstrated the possibility of learning unified representations across different tactile sensors, whereas TLA [54] and TacVLA [110] have provided early examples of contact-rich manipulation that directly combine tactile sensing and language. TaF-VLA [111] introduced a recent approach in this direction by explicitly linking tactile sensing and physical interaction through tactile-force alignment, thereby moving toward grounding tactile information in language. Tactile-VLA [112] further presented a case in which position-force hybrid control was combined with VLA. However, these studies remain limited in task scope and data scale, and standardized tactile-language datasets at the level of general VLA benchmarks are still lacking. Second, it is necessary to model, in an interpretable manner, how such contact-level concepts intervene in action generation. Future research should therefore extend the manipulation semantics that vision-centered VLA fails to capture through tactile-language alignment, force-aware instruction grounding, synthetic tactile data generation, sim-to-real transfer, and dynamic grounding mechanisms.

### 6.3. Asynchronous and Multi-Rate Sensor Fusion Frameworks

In real robotic systems, sensors do not operate at the same frequency. Camera perception and language-based inference are typically performed at relatively low frequencies, whereas IMU, force/torque, tactile sensing, and event-based vision are updated at much higher frequencies or in an asynchronous manner. Current multi-modal VLA research has largely addressed fusion at the level of jointly providing multiple sensors as input, but the question of how to theoretically align asynchronous and multi-rate sensors remains insufficiently studied [60,100,101].

Future research should advance in at least three directions. First, a fusion theory is needed that explicitly accounts for temporal delays and update rates across sensors. Second, a multi-rate execution framework is required to connect high-frequency sensor information to low-frequency policies through summary features, event triggers, or hierarchical control structures. For example, hierarchical diffusion policy [100] proposed a structure that separates high-level planning from low-level execution, whereas SDP [60] suggested a direction for maintaining a tighter control loop by reusing partially denoised future actions. Third, from the perspective of event-triggered control, it is necessary to combine the principles of asynchronous triggering summarized by Nowzari et al. [101] with robot learning in order to build a hybrid scheduling framework that integrates periodic inference with event-based replanning. Sensor fusion should therefore no longer be treated merely as a problem of representation learning but as a problem of temporal alignment across the entire perception–action loop.

### 6.4. Real-Sensor-Grounded Benchmarks and Evaluation Protocols

As discussed in Section 5, most current representative benchmarks remain centered on RGB or RGB-D observations and tend to rely excessively on final SR as the primary performance measure [103,104,105,106,107,108]. Accordingly, one of the major challenges for future VLA research is to move beyond simulation bias and establish benchmarks and evaluation protocols that explicitly reflect real sensor configurations. Such benchmarks should satisfy at least the following requirements: First, they should define a range of sensor combinations in a comparable manner, including RGB, depth, tactile sensing, F/T, IMU, thermal, and event cameras. Second, they should explicitly specify sensor update rates and synchronization conditions so that multi-rate issues can be properly evaluated. Third, they should provide a multidimensional evaluation framework that includes not only SR but also robustness, worst-case performance, safety, inference latency, and compute cost. Fourth, they should systematically incorporate sensor ablation and perturbation settings so that it becomes possible to reveal which sensors are actually valuable under which conditions. Only when such real-sensor benchmarks are established can VLA research move beyond RGB-centric simulation optimization and progress toward a more realistic direction that reflects actual deployment environments.

### 6.5. Safe and Reliable Deployment of Sensor-Rich VLA Systems

Current VLA research is primarily evaluated in terms of average performance and generalization. However, in real deployment environments, safe responses to sensor failure and graceful performance degradation are far more critical [113,114,115]. This is particularly true in safety-critical settings such as healthcare, industrial automation, and logistics, where sensor dropout, calibration drift, camera occlusion, tactile saturation, or force sensor failure can immediately lead to hazardous situations. Even under such conditions, the system should be able to degrade safely, switch to conservative behavior when necessary, and detect failures early before they escalate.

**Failure probability, redundancy, and fail-safe design.** A safety-oriented treatment of sensor failure requires connecting modality-level faults to system-level risk. Let $p_{m}$ denote the probability that modality *m* produces an unreliable measurement within a given operational window, for example, due to occlusion, saturation, calibration drift, or hardware malfunction. Under the idealized assumption that modality failures are independent and that at least one functioning modality is sufficient for degraded operation, the probability that the system remains operational can be approximated as

(14) $$P_{operational}=1-\prod_{m=1}^{M}p_{m}$$

This formulation provides a simple rationale for sensor redundancy, but it also highlights the importance of avoiding common-cause failures. For example, combining RGB and depth sensors that both depend on optical visibility may not provide the same safety benefit as combining vision with F/T or tactile sensing. Therefore, deployment-oriented VLA systems should prefer physically diverse modalities and reliability-aware fusion mechanisms that can reduce trust in a degraded sensor rather than simply concatenating all inputs.

**Fault detection and safety standards.** Redundancy is useful only when sensor faults can be detected quickly enough to prevent unsafe actions. Relevant mechanisms include residual-based detection, which compares sensor readings with model-predicted values; model-based bounds checking, which uses known physical limits such as force thresholds or IMU ranges; and data-driven anomaly detection, which identifies deviations from normal sensor distributions. The choice of detector should account for detection latency, because high-speed contact events may require faster responses than neural anomaly detectors can provide. In industrial or human-collaborative settings, these design choices should also be interpreted in relation to established safety frameworks such as ISO 10218 [116,117] for industrial robot safety and IEC 61508 [118] for functional safety. Although formal certification is outside the scope of this survey, these standards make clear that sensor redundancy, diagnostic coverage, and fail-safe responses must be considered at the system design stage rather than treated as post hoc additions.

**Adversarial conditions and sensor spoofing.** Real-world deployment also requires considering deliberate or environmental sensor spoofing. RGB cameras may be affected by adversarial patterns or misleading visual cues, depth sensors may be disrupted by infrared interference or reflective materials, IMUs can be affected by abnormal vibration, and tactile sensors may produce misleading contact patterns under unusual surface conditions. A practical defense is cross-modal consistency checking, in which visual, depth, F/T, tactile, and proprioceptive signals are compared for physically plausible agreement. If cross-modal agreement falls below a predefined threshold, the system should enter a degraded mode, request human intervention, or execute a fail-safe stop depending on the severity of the task.

Accordingly, future VLA systems should incorporate fault tolerance as a core design objective rather than treating robustness alone as sufficient. This includes sensor reliability estimation, uncertainty-based reweighting, modality-specific fallback policies, degraded-mode control, and fail-safe switching [113,114]. From the benchmark perspective, evaluation should address not only how well a system performs under normal conditions but also how safely it fails when sensors malfunction. Recent surveys on fault-tolerant robotics and safety-oriented reviews have already emphasized this requirement [113,114,115], yet standard design guidelines that satisfy these criteria remain underdeveloped in the VLA context. Ultimately, what matters in real deployment is not merely achieving a high average success rate but whether the system can maintain reliable behavior under unexpected sensor anomalies.

As the preceding discussions suggest, the next stage of VLA research should move beyond simply connecting larger foundation models to robot control. It must instead expand toward jointly addressing sensor diversity, temporal asynchrony, contact-grounded semantic representation, safe deployment, and real-sensor evaluation frameworks. From a sensor-centered perspective, the central question for future research is no longer whether language and vision can be combined but rather how information from heterogeneous physical sensors can be unified into a common representation and used robustly and safely within an actual control loop. This survey argues that these issues should become central research challenges for future VLA and further suggests that sensor-agnostic architectures and real-sensor-grounded benchmarks will form a key foundation for the next generation of Physical AI.

## 7. Methodological Limitations of This Survey

This survey was designed as a technical, sensor-centric review rather than as a PRISMA-style systematic review or a bibliometric meta-analysis. Although the literature search scope, searched databases, representative Boolean queries, search period, and inclusion and exclusion criteria are specified in the section Introduction, several methodological limitations should be acknowledged explicitly so that readers can interpret the coverage and reproducibility of this survey appropriately.

First, this survey did not employ independent dual-reviewer screening or a formal inter-rater agreement assessment. The selection and organization of the literature were guided by the objective of synthesizing the sensor–fusion–action design space in VLA-based robotic manipulation rather than by a fully protocol-driven systematic review process. Consequently, although the survey covers representative studies across sensor modalities, data collection pipelines, fusion strategies, action representations, and evaluation protocols, it may not exhaustively include all relevant publications.

Second, a PRISMA flow diagram was not used, and this survey therefore does not report a formal count of records identified, screened, excluded, and finally included. This choice reflects the technical-survey orientation of the paper, which aims to establish a conceptual and design-oriented framework for sensor-centric VLA research rather than to conduct a quantitative evidence synthesis.

Third, although representative Boolean queries and database sources are reported in the section Introduction to improve reproducibility, exact reproducibility remains limited because search interfaces differ across IEEE Xplore, ACM Digital Library, arXiv, Google Scholar, and publisher databases. In addition, given the rapid pace of development in VLA and Physical AI, recently emerging preprints and concurrent works may not be fully captured.

These limitations do not undermine the main purpose of this survey, which is to identify and organize the underexplored role of sensing, multi-modal fusion, and sensor-to-action alignment in VLA-based robotic manipulation. Future work could complement this technical survey with a PRISMA-compliant systematic review that incorporates independent multi-reviewer screening and quantitative bibliometric analysis.

## 8. Conclusions

This survey reviewed Vision–Language–Action models for robotic manipulation from a sensor–fusion–action perspective. The main finding is that the performance and deployability of VLA systems are not determined only by model scale, backbone architecture, or action decoder design but also by the physical properties of sensor modalities, the reliability of calibration and synchronization, the structure of multi-modal fusion, and the temporal coupling between sensor updates and action generation.

Through this analysis, the survey organized major sensor modalities, data collection pipelines, fusion strategies, action representations, benchmarks, and evaluation metrics into a unified design space. It further showed that RGB-centric inputs and single success-rate-based evaluation are insufficient for characterizing sensor-rich, contact-rich, and deployment-oriented manipulation. Overall, this survey highlights the need to evaluate VLA systems as integrated perception–fusion–action pipelines rather than as model architectures alone.

## Figures and Tables

**Table 1:** Comparison between representative prior surveys and this work across major analytical dimensions of VLA research. ✓ = covered as a primary axis; △ = partially or peripherally covered; ✗ = not covered as a structured analysis axis.

Survey	Main Focus	Sensor Modality Taxonomy	Failure Mode Analysis	Calibration and Synchronization	Data Collection/Data Engine	Fusion Strategy Analysis	Evaluation Framework	Sensor-Centric Perspective
Ma et al. (2024) [22]	Broad VLA/embodied AI overview	✗	✗	✗	△	✗	△	✗
Zhong et al. (2025) [23]	Action token taxonomy	✗	✗	✗	△	✗	△	✗
Din et al. (2025) [21]	Architectures, datasets, and simulators	✗	✗	✗	✓	△	✓	✗
Shao et al. (2025) [24]	Large-VLM-based VLA architectures	✗	✗	✗	△	✗	△	✗
Li et al. (2025) [25]	Structures, datasets, pre/post-training, and evaluation	✗	✗	✗	△	✗	✓	✗
Wang et al. (2026) [26]	Data-centric VLA survey	✗	✗	✗	✓	✗	✓	✗
Han et al. (2026) [27]	Robotic vision, multi-modal fusion, and VLMs	△	✗	✗	✓	✓	△	△
Ours	Sensor–fusion–action for robotic manipulation	✓	✓	✓	✓	✓	✓	✓

**Table 2:** Comparison of representative vision sensors for VLA systems in terms of sensing role, use in VLA, and practical limitations. The role of these vision sensors within the broader VLA pipeline is illustrated in Figure 1.

Sensor	Sensing Role	Typical Use in VLA	Key Limitations
RGB Camera [2,10,11,32,33]	Rich semantic and appearance information for object recognition, scene understanding, and language grounding	Primary visual input for most VLA models; encoded by vision backbones and fused with language tokens	No direct depth information; sensitive to occlusion, illumination changes, reflections, and transparent objects
Depth/RGB-D Sensor [34,35,36,37]	Geometric structure, distance, and surface shape for grasping and contact-aware planning	Combined with RGB to support 3D scene understanding, grasp pose estimation, and manipulation planning	Failure on transparent/reflective surfaces; reduced reliability under strong ambient light; alignment with pre-trained VLM tokens remains nontrivial
Event Camera [39,40,41,42]	High temporal resolution and high dynamic range for fast motion and dynamic scenes	Potential input for motion-sensitive manipulation and high-speed perception, often after conversion to event frames or related representations	Sparse asynchronous output; limited large-scale pre-training data; weak compatibility with standard RGB-based VLM pipelines an open problem
Fisheye/Wide-angle Camera [6,46,47,48]	Wide field of view for workspace coverage and reduced blind spots	Useful for mobile manipulation or long-horizon tasks requiring large scene coverage	Severe radial distortion; weaker compatibility with perspective-camera-pre-trained encoders; rectification may still leave residual distortion

**Table 3:** Case-dependent tolerability of additional sensing sources in sensor-rich VLA systems. The judgment in the last column should be read together with the multidimensional evaluation metrics in Section 5.

Task/Condition	Added Sensing Source	Expected Benefit	Main Overhead	Tolerability Judgment
Simple rigid-object pick-and-place under good visibility	Depth or tactile	Limited improvement over RGB and proprioception	Added preprocessing and synchronization	Often not necessary unless the failure rate is high
Transparent or reflective object manipulation	Depth, tactile, or F/T	Compensates for unreliable RGB or depth cues and improves contact estimation	Calibration and fusion latency	Often tolerable when visual uncertainty causes failures
Contact-rich insertion or assembly	Tactile or F/T	Detects contact, jamming, slip, and excessive force	High-rate sensing and control-loop integration	Usually tolerable, especially for safety and robustness
Deformable-object manipulation	Tactile, F/T, or multi-view vision	Improves deformation and contact-state estimation	Increased sensor bandwidth and fusion complexity	Tolerable when deformation state cannot be inferred visually
Fast motion or mobile manipulation	IMU or event camera	Provides high-rate motion cues and reduces visual latency	Multi-rate alignment and asynchronous fusion	Tolerable if summarized or event-triggered efficiently
Industrial monitoring or diagnostic operation	Thermal, vibration, acoustic, or IMU sensing	Provides failure, anomaly, or quality-control information	Additional inference or monitoring cost	Tolerable if used as supervisory feedback rather than high-frequency policy input

**Table 4:** Comparison of representative VLA training datasets in terms of sensor configuration and data collection methodology. Modality availability may vary within Open X-Embodiment because it aggregates heterogeneous multi-institution datasets and within MimicGen because it depends on the underlying simulator and task configuration. BridgeData V2 and DROID include language annotations, whereas RLBench additionally provides segmentation masks.

Dataset	RGB	Depth	Tactile	F/T	Proprio	Collection Method	Scale/Note
Open X-Embodiment [17]	✓	Varies	Varies	Varies	✓	Multi-institution real-world demonstrations	1M+ trajectories; 22 robot embodiments
DROID [46]	✓	✓	✗	✗	✓	In-the-wild teleoperation/human demonstrations	~76k trajectories; 350 h; language annotations included
ALOHA [59]	✓	✗	✗	✗	✓	Whole-body bimanual teleoperation	~50 demonstrations per task in the reported setup
BridgeData V2 [72]	✓	✓	✗	✗	✓	VR teleoperation + scripted rollouts	60k trajectories
MimicGen [80]	Sim dependent	Sim dependent	Sim dependent	Sim dependent	✓	Simulation-based generation from a small number of human demonstrations	50k+ generated demos from <200 human demos across 18 tasks
Mobile ALOHA [83]	✓	✗	✗	✗	✓	Whole-body mobile manipulation teleoperation	~50 demonstrations per task; includes mobile base velocity in proprioception
RH20T [81]	✓	✓	(limited)	✓	✓	Contact-rich teleoperation	~110k sequences; some sequences include fingertip tactile sensing
RLBench [82]	✓	✓	✗	✗	✓	Scripted/motion-planned simulation	100 tasks; effectively unlimited demonstrations

**Table 5:** Computational comparison of fusion strategies. A graphical overview of these fusion strategies, together with reliability weighting and action representation, is provided in Figure 4.

Strategy	Main Operation	Cost Trend	Strength	Limitation
Early fusion	Joint self-attention over all modality tokens	$O(L^{2}d)$	Dense cross-modal interaction	High memory and latency
Late fusion	Independent encoding followed by vector-level fusion	O(Md) after summarization	Modular and efficient	Possible loss of low-level interactions
Hybrid fusion	Cross-attention between selected modality pairs	$O(L_{q}L_{kv}d)$ per interaction	Controlled cross-modal reasoning	Design complexity and pair selection

**Table 6:** Metric-level summary of evaluation criteria for sensor-rich VLA systems. Occurrence is reported qualitatively, rather than as a percentage-based frequency count, because the reviewed works differ substantially in task scope, benchmark protocol, sensor configuration, number of trials, and reporting granularity.

Category	Metric/Formula	Meaning	Pros	Cons/Limitation	Occurrence
Task completion	SR = successful trials/total trials	Whether the task is completed	Simple, intuitive, and easy to compare	Hides failure cause, safety, and sensor contribution	Common
Task progress	Sub-goal completion = completed sub-goals/total sub-goals	Partial progress in long-horizon tasks	More informative than binary SR	Needs sub-goal annotation	Partial
Robustness	RSR = mean SR under perturbation/OOD/sensor noise	Average performance under domain shift	Captures stability beyond clean settings	Depends on perturbation protocol	Partial
Worst-case robustness	WSR = min SR across perturbation conditions	Lower-bound performance in the hardest condition	Reveals edge-case failures	Sensitive to perturbation-set design	Emerging
Safety	Collision count, force-limit violation, unsafe-contact events	Whether the policy avoids hazardous behavior	Critical for real deployment	Needs safety sensors or manual labels	Emerging
Contact stability	Slip count, regrasp frequency, excessive-force events	Grasp and contact reliability	Useful for tactile/F/T manipulation	Needs tactile/F/T sensing	Emerging
Execution quality	Smoothness, jerk, corrective-action frequency	Physical stability and control quality	Captures quality beyond success/failure	Hardware and controller dependent	Partial
Real-time feasibility	AIL = mean inference time per step or episode	Inference-time burden	Important for real-time deployment	Hardware/implementation dependent	Partial
End-to-end latency	Policy-loop latency = sensing + preprocessing + fusion + inference + actuation	Full perception–action delay	More deployment-relevant than inference only	Requires system-level profiling	Emerging
Efficiency/cost	Compute cost, memory, energy; CpS = cost/successful trial	Resource consumption per success	Links performance with deployment cost	Platform and definition dependent	Emerging
Sensor contribution	Ablation drop = SR_full − SR_without modality	Contribution of a sensor modality	Directly supports sensor selection	Needs controlled ablation	Emerging
Failure diagnosis	Failure-type distribution (perception, calibration, contact, planning, and execution)	Dominant failure sources	Useful for system improvement	Needs detailed failure labeling	Emerging

## Data Availability

Data are contained within the article.

## References

[1] Levine S., Pastor P., Krizhevsky A., Ibarz J., Quillen D. (2016). Learning Hand-Eye Coordination for Robotic Grasping with Deep Learning and Large-Scale Data Collection. arXiv.

[2] Eric J., Irpan A., Khansari M., Kappler D., Ebert F., Lynch C., Levine S., Finn C. (2022). BC-Z: Zero-Shot Task Generalization with Robotic Imitation Learning. Proceedings of the 5th Conference on Robot Learning.

[3] Zhang T., McCarthy Z., Jow O., Lee D., Chen X., Goldberg K., Abbeel P. (2017). Deep Imitation Learning for Complex Manipulation Tasks from Virtual Reality Teleoperation. arXiv.

[4] Calandra R., Owens A., Jayaraman D., Lin J., Yuan W., Malik J., Adelson E.H., Levine S. (2018). More Than a Feeling: Learning to Grasp and Regrasp using Vision and Touch. IEEE Robot. Autom. Lett..

[5] Pinto L., Gupta A. Supersizing Self-Supervision: Learning to Grasp from 50K Tries and 700 Robot Hours. Proceedings of the 2016 IEEE International Conference on Robotics and Automation (ICRA).

[6] Tirado-Garin A., Civera J. (2025). AnyCalib: On-Manifold Learning for Model-Agnostic Single-View Camera Calibration. Proceedings of the IEEE/CVF International Conference on Computer Vision.

[7] Yuan W., Dong S., Adelson E.H. (2017). GelSight: High-Resolution Robot Tactile Sensors for Estimating Geometry and Force. Sensors.

[8] Mason M.T. (2018). Toward Robotic Manipulation. Annu. Rev. Control Robot. Auton. Syst..

[9] Anthony B., Brown N., Carbajal J., Chebotar Y., Dabis J., Finn C., Gopalakrishnan K., Hausman K., Herzog A., Hsu J. (2022). RT-1: Robotics Transformer for Real-World Control at Scale. arXiv.

[10] Brianna Z., Yu T., Xu S., Xu P., Xiao T., Xia F., Wu J., Wohlhart P., Welker S., Wahid A. (2023). RT-2: Vision-Language-Action Models Transfer Web Knowledge to Robotic Control. Conference on Robot Learning.

[11] Kim M.J., Pertsch K., Karamcheti S., Xiao T., Balakrishna A., Nair S., Rafailov R., Foster E., Lam G., Sanketi P. (2024). OpenVLA: An Open-Source Vision-Language-Action Model. arXiv.

[12] Reed S., Zolna K., Parisotto E., Colmenarejo S.G., Novikov A., Barth-Maron G., Gimenez M., Sulsky Y., Kay J., Springenberg J.T. (2022). A Generalist Agent (Gato). arXiv.

[13] Chi C., Xu Z., Feng S., Cousineau E., Du Y., Burchfiel B., Tedrake R., Song S. (2024). Diffusion policy: Visuomotor policy learning via action diffusion. Int. J. Robot. Res..

[14] Lipman Y., Chen R.T.Q., Ben-Hamu H., Nickel M., Le M. Flow Matching for Generative Modeling. Proceedings of the 11th International Conference on Learning Representations (ICLR 2023).

[15] Ghosh D., Walke H., Pertsch K., Black K., Mees O., Dasari S., Hejna J., Kreiman T., Xu C., Luo J. Octo: An Open-Source Generalist Robot Policy. Proceedings of the Robotics: Science and Systems (RSS).

[16] Vaswani A., Shazeer N., Parmar N., Uszkoreit J., Jones L., Gomez A.N., Kaiser Ł., Polosukhin I. Attention is all you need. Proceedings of the 31st International Conference on Neural Information Processing Systems (NIPS 2017).

[17] O’Neill A., Rehman A., Maddukuri A., Gupta A., Padalkar A., Lee A., Pooley A., Gupta A., Mandlekar A., Jain A. Open X-Embodiment: Robotic Learning Datasets and RT-X Models. Proceedings of the 2024 IEEE International Conference on Robotics and Automation (ICRA).

[18] Kevin B., Brown N., Driess D., Esmail A., Equi M., Finn C., Fusai N., Groom L., Hausman K., Ichter B. (2024). π_0_: A Vision-Language-Action Flow Model for General Robot Control. arXiv.

[19] Li X., Liu M., Zhang H., Yu C., Xu J., Wu H., Kong T. (2023). Vision-Language Foundation Models as Effective Robot Imitators (RoboFlamingo). arXiv.

[20] Awadalla A., Gao I., Gardner J., Hessel J., Hanafy Y., Zhu W., Marathe K., Bitton Y., Gadre S., Sagawa S. (2023). OpenFlamingo: An Open-Source Framework for Training Large Autoregressive Vision-Language Models. arXiv.

[21] Ud Din M., Akram W., Saad Saoud L., Rosell J., Hussain I. (2025). Vision Language Action Models in Robotic Manipulation: A Systematic Review. arXiv.

[22] Ma Y., Song Z., Zhuang Y., Hao J., King I. (2024). A Survey on Vision-Language-Action Models for Embodied AI. arXiv.

[23] Zhong Y., Bai F., Cai S., Huang X., Chen Z., Zhang X., Wang Y., Guo S., Guan T., Lui K.N. (2025). A Survey on Vision-Language-Action Models: An Action Tokenization Perspective. arXiv.

[24] Shao R., Li W., Zhang L., Zhang R., Liu Z., Chen R., Nie L. (2025). Large VLM-based Vision-Language-Action Models for Robotic Manipulation: A Survey. arXiv.

[25] Li H., Chen Y., Cui W., Liu W., Liu K., Zhou M., Zhang Z., Zhao D. (2025). Survey of Vision-Language-Action Models for Embodied Manipulation. arXiv.

[26] Wang Z., Wang B., Zhang H., Du T., Chen T., Sun G., He Y., Shen Z., Ye W., Li A. (2026). Vision-Language-Action in Robotics: A Survey of Datasets, Benchmarks, and Data Engines. arXiv.

[27] Han X., Chen S., Fu Z., Feng Z., Fan L., An D., Wang C., Guo L., Meng W., Zhang X. (2026). Multimodal Fusion and Vision–Language Models: A Survey for Robot Vision. Inf. Fusion.

[28] Krizhevsky A., Sutskever I., Hinton G.E. (2012). ImageNet classification with deep convolutional neural networks. Advances in Neural Information Processing Systems 25 (NIPS 2012).

[29] Palazzo L., Suglia V., Grieco S., Buongiorno D., Pagano G., Bevilacqua V., D’Addio G. Optimized Deep Learning-Based Pathological Gait Recognition Explored Through Network Analysis of Inertial Data. Proceedings of the 2025 IEEE Medical Measurements & Applications (MeMeA).

[30] Buongiorno D., Prunella M., Grossi S., Hussain S.M., Rennola A., Longo N., Di Stefano G., Bevilacqua V., Brunetti A. (2022). Inline Defective Laser Weld Identification by Processing Thermal Image Sequences with Machine and Deep Learning Techniques. Appl. Sci..

[31] Dosovitskiy A., Beyer L., Kolesnikov A., Weissenborn D., Zhai X., Unterthiner T., Dehghani M., Minderer M., Heigold G., Gelly S. (2020). An Image is Worth 16×16 Words: Transformers for Image Recognition at Scale. arXiv.

[32] Ahn M., Brohan A., Brown N., Chebotar Y., Cortes O., David B., Finn C., Gopalakrishnan K., Hausman K., Herzog A. (2023). Do As I Can, Not As I Say: Grounding Language in Robotic Affordances. Proceedings of the 6th Conference on Robot Learning (CoRL).

[33] Driess D., Xia F., Sajjadi M.S.M., Lynch C., Chowdhery A., Ichter B., Wahid A., Tompson J., Vuong Q., Yu T. (2023). PaLM-E: An Embodied Multimodal Language Model. arXiv.

[34] Wu T., Jing Y., Cheang C., Chen G., Xu J., Li X., Liu M., Li H., Kong T. GR-1: Unleashing Large-Scale Video Generative Pre-Training for Visual Robot Manipulation. Proceedings of the International Conference on Learning Representations.

[35] Bharadhwaj H., Vakil J., Sharma M., Gupta A., Tulsiani S., Kumar V. RoboAgent: Generalization and Efficiency in Robot Manipulation via Semantic Augmentation and Action Chunking. Proceedings of the 2024 IEEE International Conference on Robotics and Automation (ICRA).

[36] Zhao H., Jiang L., Fu C.-W., Jia J. Point Transformer. Proceedings of the IEEE/CVF International Conference on Computer Vision (ICCV).

[37] Yu X., Tang L., Rao Y., Huang T., Zhou J., Lu J. (2021). Point-BERT: Pre-Training 3D Point Cloud Transformers with Masked Point Modeling. arXiv.

[38] Shridhar M., Manuelli L., Fox D. (2022). PerAct: Perceiver-Actor for Robotics Manipulation. arXiv.

[39] Gallego G., Delbrück T., Orchard G., Bartolozzi C., Taba B., Censi A., Leutenegger S., Davison A.J., Conradt J., Daniilidis K. (2022). Event-Based Vision: A Survey. IEEE Trans. Pattern Anal. Mach. Intell..

[40] Lichtsteiner P., Posch C., Delbrück T. (2008). A 128×128 120 dB 15 μs Latency Asynchronous Temporal Contrast Vision Sensor. IEEE J. Solid-State Circuits.

[41] Rebecq H., Ranftl R., Koltun V., Scaramuzza D. (2019). Events-to-Video: Bringing Modern Computer Vision to Event Cameras. Proceedings of the IEEE/CVF Conference on Computer Vision and Pattern Recognition.

[42] Sun Z., Messikommer N., Gehrig D., Scaramuzza D., Avidan S., Brostow G., Cissé M., Farinella G.M., Hassner T. (2022). ESS: Learning Event-Based Semantic Segmentation from Still Images. Computer Vision—ECCV 2022, Lecture Notes in Computer Science.

[43] Gehrig M., Aarents W., Gehrig D., Scaramuzza D. (2021). DSEC: A Stereo Event Camera Dataset for Driving Scenarios. IEEE Robot. Autom. Lett..

[44] Radford A., Kim J.W., Hallacy C., Ramesh A., Goh G., Agarwal S., Sastry G., Askell A., Mishkin P., Clark J. Learning Transferable Visual Models from Natural Language Supervision. Proceedings of the 38th International Conference on Machine Learning (ICML 2021).

[45] Wu Z., Liu X., Gilitschenski I. (2023). EventCLIP: Adapting CLIP for Event-Based Vision. arXiv.

[46] Khazatsky A., Pertsch K., Nair S., Balakrishna A., Dasari S., Karamcheti S., Nasiriany S., Srirama M.K., Chen L.Y., Ellis K. (2024). DROID: A Large-Scale In-The-Wild Robot Manipulation Dataset. arXiv.

[47] Shafiullah N.M.M., Rai A., Etukuru H., Liu Y., Misra I., Chintala S., Pinto L. (2023). On Bringing Robots Home. arXiv.

[48] Dal Cin A., Dikov G., Ju J., Ghafoorian M. (2025). AnyMap: Learning a General Camera Model for Structure-from-Motion with Unknown Cameras. Proceedings of the IEEE/CVF Conference on Computer Vision and Pattern Recognition (CVPR).

[49] Frisoli A., Leonardis D. (2024). Wearable Haptics for Virtual Reality and Beyond. Nat. Rev. Electr. Eng..

[50] Bortone I., Barsotti M., Leonardis D., Crecchi A., Tozzini A., Bonfiglio L., Frisoli A. (2020). Immersive Virtual Environments and Wearable Haptic Devices in Rehabilitation of Children with Neuromotor Impairments: A Single-Blind Randomized Controlled Crossover Pilot Study. J. NeuroEng. Rehabil..

[51] Calandra R., Owens A., Upadhyaya M., Yuan W., Lin J., Adelson E.H., Levine S. (2017). The Feeling of Success: Does Touch Sensing Help Predict Grasp Outcomes?. arXiv.

[52] Dong S., Yuan W., Adelson E.H. (2017). Improved GelSight Tactile Sensor for Measuring Geometry and Slip. Proceedings of the 2017 IEEE/RSJ International Conference on Intelligent Robots and Systems (IROS).

[53] Lambeta M., Chou P.-W., Tian S., Yang B., Maloon B., Most V.R., Stroud D., Santos R., Byagowi A., Kammerer G. (2020). DIGIT: A Novel Design for a Low-Cost Compact High-Resolution Tactile Sensor with Application to In-Hand Manipulation. IEEE Robot. Autom. Lett..

[54] Hao P., Zhang C., Li D., Cao X., Hao X., Cui S., Wang S. (2025). TLA: Tactile-Language-Action Model. arXiv.

[55] Bi J., Ma K.Y., Hao C., Zheng M.S., Soh H. (2025). VLA-Touch: Enhancing Vision-Language-Action Models with Dual-Level Tactile Feedback. arXiv.

[56] Liu W., Wang J., Wang Y., Wang W., Lu C. (2024). ForceMimic: Force-Centric Imitation Learning with Force-Motion Capture System for Contact-Rich Manipulation. arXiv.

[57] Suglia V., Palazzo L., Bevilacqua V., Passantino A., Pagano G., D’Addio G. (2024). A Novel Framework Based on Deep Learning Architecture for Continuous Human Activity Recognition with Inertial Sensors. Sensors.

[58] Jaramillo I.E., Jeong J.G., Lopez P.R., Lee C.-H., Kang D.-Y., Ha T.-J., Oh J.-H., Jung H., Lee J.H., Lee W.H. (2022). Real-Time Human Activity Recognition with IMU and Encoder Sensors in Wearable Exoskeleton Robot via Deep Learning Networks. Sensors.

[59] Zhao T.Z., Kumar V., Levine S., Finn C. Learning Fine-Grained Bimanual Manipulation with Low-Cost Hardware. Proceedings of the Robotics: Science and Systems (RSS) XIX.

[60] Høeg S.H., Du Y., Egeland O. (2024). Streaming diffusion policy: Fast policy synthesis with variable noise diffusion models. arXiv.

[61] Ahn D., Jung C., Baek J., Yoo S., Ko B.C. (2026). Shifted flow policy: Uncertainty-aware time reparameterization for visuomotor learning. Proceedings of the IEEE International Conference on Robotics and Automation (ICRA).

[62] Shrivastava A., Gangani K., Jain L., Goel M., Batra N. (2026). ThermEval: A Structured Benchmark for Evaluation of Vision-Language Models on Thermal Imagery. arXiv.

[63] Signoroni A., Savardi M., Baronio A., Benini S. (2019). Deep Learning Meets Hyperspectral Image Analysis: A Multidisciplinary Review. J. Imaging.

[64] Murray R.M., Li Z., Sastry S.S. (1994). A Mathematical Introduction to Robotic Manipulation.

[65] Lynch K.M., Park F.C. (2017). Modern Robotics: Mechanics, Planning, and Control.

[66] Furgale P., Rehder J., Siegwart R. (2013). Unified Temporal and Spatial Calibration for Multi-Sensor Systems. Proceedings of the 2013 IEEE/RSJ International Conference on Intelligent Robots and Systems.

[67] Taylor Z., Nieto J. (2016). Motion-Based Calibration of Multimodal Sensor Extrinsics and Timing Offset Estimation. IEEE Trans. Robot..

[68] Koide K., Menegatti E. (2019). General Robot-Camera Synchronization Based on Reprojection Error Minimization. Proceedings of the ARW & OAGM Workshop 2019.

[69] Koide K., Menegatti E. (2019). General Hand–Eye Calibration Based on Reprojection Error Minimization. IEEE Robot. Autom. Lett..

[70] Ha J. (2016). Probabilistic Framework for Hand-Eye and Robot-World Calibration AX=YB. IEEE Trans. Robot..

[71] Pachtrachai K., Vasconcelos F., Edwards P., Stoyanov D. (2021). Learning to Calibrate—Estimating the Hand-eye Transformation without Calibration Objects. IEEE Robot. Autom. Lett..

[72] Walke H.R., Black K., Zhao T.Z., Vuong Q., Zheng C., Hansen-Estruch P., He A.W., Myers V., Kim M.J., Du M. (2023). BridgeData V2: A Dataset for Robot Learning at Scale. Proceedings of The 7th Conference on Robot Learning.

[73] Porcini F., Chiaradia D., Marcheschi S., Solazzi M., Frisoli A. Evaluation of an Exoskeleton-Based Bimanual Teleoperation Architecture with Independently Passivated Slave Devices. Proceedings of the 2020 IEEE International Conference on Robotics and Automation (ICRA).

[74] Palagi M., Santamato G., Chiaradia D., Gabardi M., Marcheschi S., Solazzi M., Frisoli A., Leonardis D. (2023). A Mechanical Hand-Tracking System with Tactile Feedback Designed for Telemanipulation. IEEE Trans. Haptics.

[75] Nair S., Rajeswaran A., Kumar V., Finn C., Gupta A. (2023). R3M: A Universal Visual Representation for Robot Manipulation. Proceedings of the 6th Conference on Robot Learning.

[76] Grauman K., Westbury A., Byrne E., Chavis Z., Furnari A., Girdhar R., Hamburger J., Jiang H., Liu M., Liu X. Ego4D: Around the World in 3000 Hours of Egocentric Video. Proceedings of the IEEE/CVF Conference on Computer Vision and Pattern Recognition (CVPR).

[77] Chen A.S., Nair S., Finn C. (2021). Learning Generalizable Robotic Reward Functions from In-The-Wild Human Videos. arXiv.

[78] Makoviychuk V., Wawrzyniak L., Guo Y., Lu M., Storey K., Macklin M., Hoeller D., Rudin N., Allshire A., Handa A. (2021). Isaac gym: High performance gpu-based physics simulation for robot learning. arXiv.

[79] Mayank M., Roth P., Tigue J., Richard A., Zhang O., Du P., Serrano-Muñoz A., Yao X., Zurbrügg R., Rudin N. (2025). Isaac Lab: A GPU-Accelerated Simulation Framework for Multi-Modal Robot Learning. arXiv.

[80] Mandlekar A., Nasiriany S., Wen B., Akinola I., Narang Y., Fan L., Zhu Y., Fox D. (2023). MimicGen: A Data Generation System for Scalable Robot Learning using Human Demonstrations. arXiv.

[81] Fang H.-S., Fang H., Tang Z., Liu J., Wang C., Wang J., Zhu H., Lu C. RH20T: A Comprehensive Robotic Dataset for Learning Diverse Skills in One-Shot. Proceedings of the 2024 IEEE International Conference on Robotics and Automation (ICRA).

[82] James S., Ma Z., Arrojo D.R., Davison A.J. (2020). RLBench: The Robot Learning Benchmark & Learning Environment. IEEE Robot. Autom. Lett..

[83] Fu Z., Zhao T.Z., Finn C. Mobile ALOHA: Learning Bimanual Mobile Manipulation with Low-Cost Whole-Body Teleoperation. Proceedings of the 7th Annual Conference on Robot Learning (CoRL 2023).

[84] Merriaux P., Dupuis Y., Boutteau R., Vasseur P., Savatier X. (2017). A Study of Vicon System Positioning Performance. Sensors.

[85] Wang J., Olson E. AprilTag 2: Efficient and Robust Fiducial Detection. Proceedings of the 2016 IEEE/RSJ International Conference on Intelligent Robots and Systems (IROS).

[86] Garrido-Jurado S., Muñoz-Salinas R., Madrid-Cuevas F.J., Marín-Jiménez M.J. (2014). Automatic Generation and Detection of Highly Reliable Fiducial Markers under Occlusion. Pattern Recognit..

[87] Wen B., Yang W., Kautz J., Birchfield S. FoundationPose: Unified 6D Pose Estimation and Tracking of Novel Objects. Proceedings of the IEEE/CVF Conference on Computer Vision and Pattern Recognition (CVPR).

[88] Wen B., Tremblay J., Blukis V., Tyree S., Müller T., Evans A., Fox D., Kautz J., Birchfield S. BundleSDF: Neural 6-DoF Tracking and 3D Reconstruction of Unknown Objects. Proceedings of the IEEE/CVF Conference on Computer Vision and Pattern Recognition (CVPR).

[89] Moya Rueda F., Grzeszick R., Fink G.A., Feldhorst S., Ten Hompel M. (2018). Convolutional Neural Networks for Human Activity Recognition Using Body-Worn Sensors. Informatics.

[90] Sheng H., Xuanqi W., Chang Z., Jiacheng W., Pingxia D., Yuwei W. (2026). AIGC video detection based on the fusion of spatial-frequency-optical flow multimodal features. J. Syst. Eng. Electron..

[91] Dang J., Zheng H., Xu X., Wang L., Hu Q., Guo Y. (2025). Adaptive sparse memory networks for efficient and robust video object segmentation. IEEE Trans. Neural Netw. Learn. Syst..

[92] Alayrac J.-B., Donahue J., Luc P., Miech A., Barr I., Hasson Y., Lenc K., Mensch A., Millican K., Reynolds M. (2022). Flamingo: A Visual Language Model for Few-Shot Learning. arXiv.

[93] Li J., Li D., Savarese S., Hoi S.C.H. BLIP-2: Bootstrapping Language-Image Pre-training with Frozen Image Encoders and Large Language Models. Proceedings of the 40th International Conference on Machine Learning (ICML 2023).

[94] Cheang C.-L., Chen G., Jing Y., Kong T., Li H., Li Y., Liu Y., Wu H., Xu J., Yang Y. (2024). GR-2: A Generative Video-Language-Action Model with Web-Scale Knowledge for Robot Manipulation. arXiv.

[95] Ma M., Ren J., Zhao L., Testuggine D., Peng X. Are Multimodal Transformers Robust to Missing Modality?. Proceedings of the IEEE/CVF Conference on Computer Vision and Pattern Recognition (CVPR).

[96] Bednarek P., Kicki P., Walas K. (2020). On Robustness of Multi-Modal Fusion—Robotics Perspective. Electronics.

[97] Huang H., Du B. (2025). Deep Evidential Fusion with Uncertainty Quantification and Reliability Learning for Multimodal Medical Image Segmentation. Inf. Fusion.

[98] Yu S., Wang J., Hussein W., Hung P.C.K. (2024). Robust Multimodal Federated Learning for Incomplete Modalities. Comput. Commun..

[99] Li Z., Gao Y., Xing J., Cui L., Wang X. (2025). Adaptive Multi-Scale Attention for Robust Visual–Tactile Feature Fusion. IFAC-Pap..

[100] Ma X., Patidar S., Haughton I., James S. Hierarchical Diffusion Policy for Kinematics-Aware Multi-Task Robotic Manipulation. Proceedings of the IEEE/CVF Conference on Computer Vision and Pattern Recognition (CVPR).

[101] Nowzari C., Garcia E., Cortés J. (2019). Event-Triggered Communication and Control of Networked Systems for Multi-Agent Consensus. Automatica.

[102] Dong A., Starr A., Zhao Y. (2025). End-to-End Identification of Autoregressive with Exogenous Input (ARX) Models Using Neural Networks. Mach. Intell. Res..

[103] Liu B., Zhu Y., Gao C., Feng Y., Liu Q., Zhu Y., Stone P. (2023). LIBERO: Benchmarking Knowledge Transfer for Lifelong Robot Learning. Advances in Neural Information Processing Systems (NeurIPS) Datasets and Benchmarks Track.

[104] Srivastava S., Li C., Lingelbach M., Martín-Martín R., Xia F., Vainio K., Lian Z., Gokmen C., Buch S., Liu K. BEHAVIOR: Benchmark for Everyday Household Activities in Virtual, Interactive, and Ecological Environments. Proceedings of the 5th Conference on Robot Learning (CoRL 2021).

[105] Li C., Xia F., Martín-Martín R., Lingelbach M., Srivastava S., Shen B., Vainio K., Gokmen C., Dharan G., Jain T. BEHAVIOR-1K: A Benchmark for Embodied AI with 1,000 Everyday Activities and Realistic Simulation. Proceedings of the 6th Annual Conference on Robot Learning (CoRL 2022).

[106] Zhang S., Xu Z., Liu P., Yu X., Li Y., Gao Q., Fei Z., Yin Z., Wu Z., Jiang Y.-G. VLABench: A Large-Scale Benchmark for Language-Conditioned Robotics Manipulation with Long-Horizon Reasoning Tasks. Proceedings of the IEEE/CVF International Conference on Computer Vision (ICCV).

[107] Li X., Hsu K., Gu J., Pertsch K., Mees O., Walke H.R., Fu C., Lunawat I., Sieh I., Kirmani S. Evaluating Real-World Robot Manipulation Policies in Simulation. Proceedings of the 8th Annual Conference on Robot Learning (CoRL 2024).

[108] Liu M., Sheng J., Li P., Wang Z., Xu T., Xu T., Liu H. (2026). Trustworthy Evaluation of Robotic Manipulation: A New Benchmark and AutoEval Methods. arXiv.

[109] Yang F., Feng C., Chen Z., Park H., Wang D., Dou Y., Zeng Z., Chen X., Gangopadhyay R., Owens A. Binding Touch to Everything: Learning Unified Multimodal Tactile Representations. Proceedings of the IEEE/CVF Conference on Computer Vision and Pattern Recognition (CVPR).

[110] Zhang K., Zhang H., Xu Z., Zhang Z., Prince M.R.I., Li X., Han X., Zhou Y., Ajoudani A., She Y. (2026). TacVLA: Contact-Aware Tactile Fusion for Robust Vision-Language-Action Manipulation. arXiv.

[111] Huang Y., Lin P., Li W., Li D., Li J., Jiang J., Xiao C., Jiao Z. (2026). TaF-VLA: Tactile-Force Alignment in Vision-Language-Action Models for Force-aware Manipulation. arXiv.

[112] Huang J., Wang S., Lin F., Hu Y., Wen C., Gao Y. (2025). Tactile-VLA: Unlocking Vision-Language-Action Model’s Physical Knowledge for Tactile Generalization. arXiv.

[113] Visinsky M.L., Cavallaro J.R., Walker I.D. (1994). Robotic Fault Detection and Fault Tolerance: A Survey. Reliab. Eng. Syst. Saf..

[114] Khan Z., Nasir A., Mekid S. (2025). Fault-Tolerant Control Strategies for Industrial Robots: State of the Art and Future Perspective on AI-Based Fault Management. Artif. Intell. Rev..

[115] Samarathunga S.M.B.P.B., Valori M., Legnani G., Fassi I. (2025). Assessing Safety in Physical Human–Robot Interaction in Industrial Settings: A Systematic Review of Contact Modelling and Impact Measuring Methods. Robotics.

[116] (2011). Robots and Robotic Devices—Safety Requirements for Industrial Robots—Part 1: Robots.

[117] (2011). Robots and Robotic Devices—Safety Requirements for Industrial Robots—Part 2: Robot Systems and Integration.

[118] (2010). Functional Safety of Electrical/Electronic/Programmable Electronic Safety-related Systems—Part 1: General Requirements..

